# Systematic analysis of homicidal poisonings in Germany

**DOI:** 10.1007/s00210-025-04823-3

**Published:** 2025-12-13

**Authors:** Madeleine Ohm, Roland Seifert

**Affiliations:** Institute of Pharmacology, Hannover Medical School, Carl-Neuberg-Str. 1, D-30625 Hannover, Germany

**Keywords:** Homicidal poisoning, Crime, Drugs, Hospital safety

## Abstract

Homicidal poisonings receive considerable media attention but have so far received too little attention in research. This study therefore aimed to analyze homicidal poisonings from Germany to gain a better understanding of this complex topic and to better investigate or even prevent future cases. Online archives of German magazines and newspapers were searched and 63 cases from 1948–2023 were collected and evaluated. 70% of the reported cases were fatal. Perpetrators were more often women and the victims more often men. While the perpetrators were usually middle-aged, the victims were usually very young or very old. Homicidal poisonings have complex causes. The largest group of perpetrators were people who killed their partners due to relationship problems. The second largest group was medical personnel who poisoned patients, which is why the substances most frequently used were medications. The motives were often stress and excessive demands, the desire to exert power, or even to gain sympathy. Oral application of compounds by food and drink was the most common mode of poisoning, gastrointestinal symptoms being the most common signs of poisoning. In earlier decades, poisoning with the irreversible acetylcholine esterase inhibitor E605 was common. Narcotics and metals play important in poisoning as well, but many compound classes are used, often accessible over-the-counter or online. Medical doctors and hospital staff play an important role in the diagnosis of poisoning. In conclusion, some typical patterns of homicidal poisoning emerged, facilitating future investigations and prevention.

**Supplementary Information:**

The online version contains supplementary material available at 10.1007/s00210-025-04823-3.

## Introduction

Homicidal poisonings are defined as the killing of one person by another person with a toxic agent. It is a fascinating topic to many consumers of fictional media but cases from the real world also get a lot of attention. True crime cases are often discussed in newspapers and magazines and are popular on the internet. According to surveys, true crime is the most popular podcast genre (Meedia 2025) and three out of the top ten podcasts 2024 in Germany belong in this category (Podius 2025). In research, however, the topic of homicidal poisonings in real life has been under-represented and therefore, a systematic analysis of this often-overlooked crime is needed to receive useful information to solve future cases quicker and prevent future crimes from happening.

In this study we performed a toxicological analysis of some of the most prominent homicidal poisoning cases in Germany from 1948–2024. This includes the cases of Niels Högel, a male nurse who killed over 80 patients between 1999 and 2005 (Spiegel 2018), and Lydia L., known as the „black widow “, who killed four men to enrich herself financially (Friedrichsen 2008). Lesser-known cases were also included, resulting in a total of 63 cases analysed about many aspects such as the substance used, the form of application, the background, the circumstances and in some cases the impact it had on society.

Our hypothesis is that murders involving poisons and drugs most frequently occur at home, as violent crimes in general are also a predominantly domestic problem, with women primarily becoming victims of their partners (Bundeskriminalamt 2024). Many of the victims of domestic violence are children, in cases of poisoning, Münchhausen-by-proxy-syndrome may play a role (Dimsdale 2024). Therefore, victims of homicidal poisonings could also be primarily women and children. Since there are several poisonous plants in Germany (AOK 2024), it is also likely that they are frequently used as poison. Apart from these, there are many household chemicals that are suitable as murder weapons due to their easy availability (Schober 2025).

## Materials and methods

### Source material

The source for information about homicidal poisonings in Germany were articles from archives of reputable magazines and newspapers. Most cases were found in the online archive of *Der Spiegel*. *Der Spiegel* is a German newspaper from Hamburg. It exists since 1947 and because of that it is a great source to analyse homicidal poisonings since the German division until today (Wikipedia authors 2025). To get more data, cases from other newspapers were supplemented namely from articles from *ZEIT*, *Süddeutsche Zeitung* and *Stern* which are all reputable sources from Germany.

### Searching for cases

**Fig. 1:**
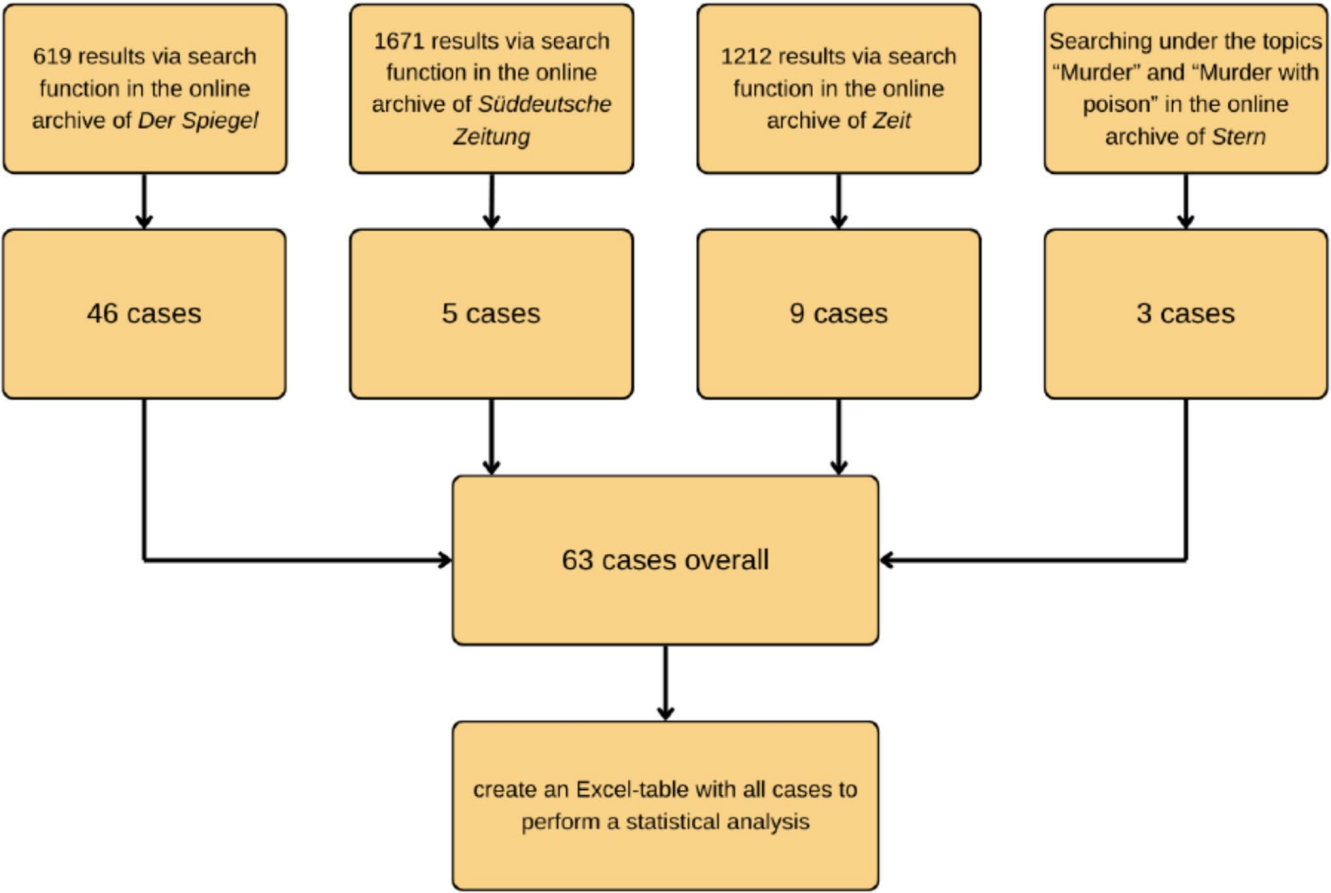
Methodical approach to finding, selecting and analysing newspaper articles about homicidal poisonings, show in a flow chart

Figure 1 shows the process of searching for cases in all four newspapers. We used a keyword search strategy to perform a case extraction from newspaper articles. With the help of searching terms like “Mord” (English “murder”), “Gift” (English “poison”) and “Medikament” (English “drug”) hundreds of articles in the online archive of *Der Spiegel* were looked through and the fitting ones were selected. After that, homicidal poisoning cases from other newspapers were added that were not found in *Der Spiegel* before. The newspapers *Süddeutsche Zeitung* and *ZEIT* also had a search function on their website that was used with the same searching terms. In the online magazine of *Stern* there was no search function but a very long list of topics which included the topics “Mord” (English “murder”) and “Giftmord” that translates to “murder with poison”. From these newspapers more articles were selected. Fig. S1 shows graphically how the cases were distributed across the four newspapers.

For the topic of homicidal poisonings a few criteria were chosen. The articles that were used as a source needed to be about a real crime case from Germany in which one person poisoned another with the intention of harming them or gaining a personal benefit from it. Cases in which a person only tried to poison someone were also included. If the perpetrator had the intention to kill the victim is irrelevant because in many cases the true intention of the perpetrator is not fully known and in some cases a court has yet to identify which legal crime took place. No crime cases from other countries were used in this analysis and no accidents or suicides. In the few cases of extended suicide the poisoning of the other person was included.

### Statistical analysis of homicidal poisonings

We performed a qualitative analysis of 63 cases of homicidal poisonings in Germany as shown in Table 1. Not all cases were solved while they were being worked on and in some cases the court case was still ongoing. Many cases contained serial killers from which some had more than ten victims. For example, out of the roughly 320 victims in total, a quarter (85) were killed by one perpetrator (Niels Högel). To prevent these cases having too much impact on the analysis one case was defined as one perpetrator and all the victims by this perpetrator fall under one case.

**Table 1:** An overview over the 63 cases of homicidal poisonings that were analyzed. Complete references are provided in the reference list

Case number	perpetrator	victim(s)	Place and year of the poisoning	Poison/drug	Mechanism of action and other information about the substance	References
1	Cardiologist (56, male)	2 patients	Berlin, 2021/2022	Propofol	Modulates the GABA_A_ receptor allosterically, has a sedative-hypnotic and respiratory depressant effect	Case: Spiegel (2023a), Ramm (2024) Propofol: Seifert (2021a), Brunton et al. (2011a)
2	Musician (62, male)	His mother, 2 colleagues	Hannover, 2022	Brodifacoum (rat poison)	Inhibits the synthesis of vitamin K-dependent coagulation factors in plasma and liver, inhibits the formation of thrombin and fibrin	Case: Spiegel (2023b) Brodifacoum: Dekant and Vamvakas (2010a)
3	Nurse (26, male)	5 patients	Munich, 2020	Sedatives, adrenaline, heparin	Sedatives could have been a barbiturate: modulates the GABA_A_ receptor allosterically, causing hyperpolarisation and inhibition of neuronal activity Adrenaline: Agonist at the alpha_x_- and beta_x_- adrenergic receptors, can trigger an increase in blood pressure, arrhythmias and myocardial infarctions Heparin: Irreversibly inhibits both thrombin and factor Xa via complex formation with antithrombin-III	Case: Spiegel (2023c) Barbiturates: Seifert (2021b), Brunton et al. (2011b) Adrenaline: Seifert (2021c) Heparin: Seifert (2021d), Brunton et al. (2011c)
4	Nurse Daniel B. (29, male)	5 patients	Homburg and Volkklingen, 2015/2016	Heart medication and sedatives	Heart medication could have been cardiac glycosides such as digoxin: a NKA inhibitor, has a positive inotropic effect by increasing the intracellular calcium concentration, produces parasympathetic stimulation with a negative dromotopic effect, can cause arrhythmias Sedatives could have been a barbiturate: modulates the GABA_A_ receptor allosterically, causing hyperpolarisation and inhibition of neuronal activity	Case: Spiegel (2022a) Digoxin: Seifert (2021e) Barbiturates: Seifert (2021b), Brunton et al. (2011b)
5	55 year old woman	Her father	Traunstein, 2022	Not known drug	Not enough information given	Case: Spiegel (2022b)
6	41 year old man	2 spouses, 1 grandmother of the spouse	Hürth, 2020/2021	Thallium	Thallium: has a toxic effect on the central and peripheral nervous system, liver, kidneys, smooth muscles of the stomach and intestines	Case: Spiegel (2021a) Thallium: Dekant and Vamvakas (2010b)
7	22 year old woman	4 year old stepdaughter	Frankenthal, 2004	Table salt	Table salt: binds water in the blood and can lead to cerebral haemorrhages	Case: Spiegel (2006a) Table Salt: Arndt and Wulf (2016)
8	unknown	25 employees of a company	Steinfeld, 2013	Rat poison	Rat poison can be either brodifacoum or thallium Brodifacoum: Inhibits the synthesis of vitamin K-dependent coagulation factors in plasma and liver, inhibits the formation of thrombin and fibrin Thallium: has a toxic effect on the central and peripheral nervous system, liver, kidneys, smooth muscles of the stomach and intestines	Case: Spiegel (2013) Brodifacoum: Dekant and Vamvakas (2010a) Thallium: Dekant and Vamvakas (2010b)
9	49 year old man	1 friend	Rheingau, 2019	Plant poison	Could have been, for example: Deadly nightshade (Atropa belladonna): competitive agonist of acetylcholine at muscarinic receptors Monkshood (Aconitine): alters the sodium channels of excitable membranes and delays repolarization	Case: Spiegel (2020a) Plant poison: Dekant and Vamvakas (2010c)
10	Nursing assistant Grzegorz W. (38, male)	12 patients	Rheingau, 2017/2018	Insulin	Insulin: increase of glucose uptake and glycogen synthesis as well as inhibition of lipolysis, hypoglycaemia	Case: Ramm (2019) Insulin: Seifert (2021f), Brunton et al. (2011e)
11	Nurse (30, female)	3 premature babies	Marburg, 2015/2016	Sedatives and anaesthetics	Sedatives could have been a barbiturate: modulates the GABA_A_ receptor allosterically, causing hyperpolarisation and inhibition of neuronal activity Anaesthetics could have been propofol: modulates the GABA_A_ receptor allosterically, has a sedative-hypnotic and respiratory depressant effect Anaesthetic could also have been a barbiturate: modulates the GABA_A_ receptor allosterically, causing hyperpolarisation and inhibition of neuronal activity	Case: Spiegel (2019a) Barbiturates: Seifert (2021b), Brunton et al. (2011b) Propofol: Seifert (2021a), Brunton et al. (2011a)
12	Nurse (25, male)	6 patients	Rheinfelden, 1975	Lanitop and Kombetin	Lanitop is a preparation of digoxin: a NKA inhibitor, has a positive inotropic effect by increasing the intracellular calcium concentration, produces parasympathetic stimulation with a negative dromotopic effect, can cause arrhythmias Kombetin is a preparation of strophanthin: also a NKA inhibitor	Case: Spiegel (1976) Digoxin: Seifert (2021e) Strophantin: Hinneburg (2015)
13	Michaela Roeder (30, female)	17 patients	Wuppertal-Barmen, 1984–1986	Catapresan and potassium chloride	Catapresan is a preparation of clonidine: alpha_2_ adrenergic receptor agonist, reduces the sympathetic tone Potassium chloride: can change the membrane potential in high doses, cells can no longer repolarize, can lead to cardiac arrest	Case: Mauz (1989) Clonidin: Seifert (2021h) Potassium chloride: Iijima (2020)
14	Midwife (33, female)	4 patients	Munich, 2014	Heparin	Heparin: irreversibly inhibits both thrombin and factor Xa via complex formation with antithrombin-III	Case: Süddeutsche Zeitung (2014) Heparin: Seifert (2021d), Brunton et al. (2011c)
15	51 year old woman	Her husband	Waldkirchen, 2018	Blood thinners	The blood thinner could have been phenprocoumon: inhibits the carboxylation and thus the function of the factors II, VII, IX and X, inhibits blood clotting	Case: Süddeutsche Zeitung (2018) Phenprocoumon: Seifert (2021i)
16	47 year old man	His wife	Thalmassing, 2020	Macumar	Macumar is a preparation of phenprocoumon: inhibits the carboxylation and thus the function of the factors II, VII, IX and X, inhibits blood clotting	Case: ZEIT (2021a) Phenprocoumon: Seifert (2021i)
17	Geriatric nurse (49, female)	2 patients	Volkach, 2020	Insulin	Insulin: increase of glucose uptake and glycogen synthesis as well as inhibition of lipolysis, hypoglycaemia	Case: ZEIT (2021a) Insulin: Seifert (2021f), Brunton et al. (2011e)
18	Nurse Irene Becker (53, female)	8 patients	Berlin, 2005/2006	Nitroprusside	Nitroprusside: non-enzymatic release of NO, sGC activation, relaxes smooth muscle cells, can lead to hypotension and cyanide intoxication	Case: Jüttner (2010) Nitroprussid: Seifert (2021j)
19	Meike W. (44, female)	At least 16 patients	Fritzlar, 2015–2018	Anaesthetics	Anaesthetics could have been Propofol: modulates the GABA_A_ receptor allosterically, has a sedative-hypnotic and respiratory depressant effect Anaesthetic could also have been a barbiturate: modulates the GABA_A_ receptor allosterically, causing hyperpolarisation and inhibition of neuronal activity	Case: Lakotta (2021) Propofol: Seifert (2021a), Brunton et al. (2011a) Barbiturates: Seifert (2021b), Brunton et al. (2011b)
20	Nurse Stephan L. (24, male)	29 patients	Sonthofen, 2003/2004	Midazolam, Etomidate, Lysthenon	Midazolam is a benzodiazepine: makes receptors more sensitive to GABA (anxiolytic, sedative-hypnotic, muscle relaxant effects), leads to respiratory depression Etomidate is a phenylethylimidazole: an allosteric GABA_A_ receptor modulator, has a sedative-hypnotic effect, no analgesia Lysthenon is the trade name of suxamethonium: a nAChR agonist, a depolarizing muscle relaxant	Case: Neumann (2004) Benzodiazepines: Seifert (2021g), Brunton et al. (2011d) Etomidate: Seifert (2021k) Suxamethonium: Seifert (2021l)
21	Nurse (52, female)	2 patients	Berlin, 2004–2006	Unknown drug	Not enough information given	Case: Spiegel (2006b)
22	Geriatric nurse (39, male)	2 patients	Bremen, 2019	Insulin, Metoprolol	Insulin: increase of glucose uptake and glycogen synthesis as well as inhibition of lipolysis, hypoglycaemia Metoprolol: a beta_1_- adrenergic receptor antagonist, has a negative chrono-, dromo- and inotropic effect	Case: Süddeutsche Zeitung (2023) Insulin: Seifert (2021f), Brunton et al. (2011e) Metoprolol: Seifert (2021m)
23	Christian F. (male)	His fiance Maria Baumer	Regensburg, 2019	Lorazepam, Tramadol	Lorazepam is a benzodiazepine: makes receptors more sensitive to GABA (anxiolytic, sedative-hypnotic, muscle relaxant effects), leads to respiratory depression Tramadol: a cyclohexanol derivative, unknown mechanism, hypotheses: a weak partial agonism at the MOR, blocks the 5-HT reuptake, antagonism at various neurotransmitter receptors, has analgesic effects, can cause respiratory distress and serotonin syndrome	Case: Ramm (2020) Benzodiazepines: Seifert (2021g), Brunton et al. (2011d) Tramadol: Seifert (2021n)
24	Nurse (35, female)	Her daughter	Hamburg, 2020	2 tranquillizers and sleeping medication	Tranquillizers could have been a barbiturate: modulates the GABA_A_ receptor allosterically, causing hyperpolarisation and inhibition of neuronal activity Sleeping medication could have been a benzodiazepine: make receptors more sensitive to GABA (anxiolytic, sedative-hypnotic, muscle relaxant effects), lead to respiratory depression	Case: Spiegel (2021b) Barbiturates: Seifert (2021b), Brunton et al. (2011b) Benzodiazepines: Seifert (2021g), Brunton et al. (2011d)
25	Nurse (47, female)	Her husband	Tegernsee, 2018	Insulin, Morphine	Insulin: increase of glucose uptake and glycogen synthesis as well as inhibition of lipolysis, hypoglycaemia Morphine: a MOR agonist, strong analgesia for four hours after intravenous administration, sedation, hypnotic-anxiolytic and antitussive effects	Case: Süddeutsche Zeitung (2020) Insulin: Seifert (2021f), Brunton et al. (2011e) Morphine: Seifert (2021o)
26	73 year old woman	Her husband	Neuperlach, 2018	unknown	Not enough information given	Bernstein (2018)
27	Farmer (53, male)	His parents	Wettstetten, 2016	rat poison	Rat poison is either brodifacoum or thallium Brodifacoum: Inhibits the synthesis of vitamin K-dependent coagulation factors in plasma and liver, inhibits the formation of thrombin and fibrin Thallium: has a toxic effect on the central and peripheral nervous system, liver, kidneys, smooth muscles of the stomach and intestines	Case: Vogler (2018) Brodifacoum: Dekant and Vamvakas (2010a) Thallium: Dekant and Vamvakas (2010b)
28	Christian K. (42, male)	His wife	Göttingen, 2014	Lead-acetate, mercury	Lead: affects the central and peripheral nervous system, haematopoietic system and the kidneys, causing nausea, intestinal colic, constipation, toxic blood count changes, liver and kidney damage, fatigue, headaches and muscle pain Mercury: pronounced corrosive effect, can cause bronchitis, bronchiolitis and even pneumonia when inhaled, can cause gastroenteritis with bloody diarrhoea, damages the gastrointestinal tract and kidneys, leads to headaches, dizziness, paralysis, tremors	Case: Eisenhardt (2015) Lead: Dekant and Vamvakas (2010d) Mercury: Dekant and Vamvakas (2010e)
29	Tanja Eimesser (31, female)	Her husband	Königsbrunn, 2017	Dormicum, Ketanest, Hypnomidate	Dormicum is a preparation of midazolam, a benzodiazepine: makes receptors more sensitive to GABA (anxiolytic, sedative-hypnotic, muscle relaxant effects), leads to respiratory depression Ketanest is esketamine: an allosteric modulator on NMDAR, has especially sedative-hypnotic and analgesic effects (dissociative anaesthesia) Hypnomidate is Etomidate, a phenylethylimidazole: an allosteric GABA_A_ receptor modulator, has a sedative-hypnotic effect, no analgesia	Case: Doinet (2007) Benzodiazepines: Seifert (2021g), Brunton et al. (2011d) Esketamine: Seifert (2021a) Etomidate: Seifert (2021k)
30	Irene Henn (29, female) and Peter Kaes (32, male)	Peter Henn (husband of Irene) and his colleague Günther Hilger	Bad Neuennahr, 1968	E605 (parathion)	E605 is parathion: an organophosphate, inhibits acetylcholinesterase, causes pulmonary oedema, respiratory and cardiac arrest	Case: Spiegel (1970) E605: Dekant and Vamvakas (2010e)
31	73 year old woman	Her husband	Munich, 2018	Antifreeze ethylene glycol	Ethylene glycol: leads to metabolic acidosis, kidney failure, can also cause calcium oxalate crystals	Case: Spiegel (2019d) Ethylene glycol: Ringler et al. (1994)
32	49 year old woman and her 44 year lover	Her husband	Bielefeld, 2022	Methadone, pregabalin, clonazepam	Methadone: a MOR agonist, is used for substitution in MOR agonist addicts Pregabalin: calcium channel blocker, blocks P/Q-type calcium channels, has antiepileptic, sedative effects, replacement drug in MOR agonist addicts Clonazepam is a benzodiazepine: makes receptors more sensitive to GABA (anxiolytic, sedative-hypnotic, muscle relaxant effects), leads to respiratory depression	Case: ZEIT (2023) Methadone: Seifert (2021n) Pregabalin: Seifert (2021p) Benzodiazepines: Seifert (2021g), Brunton et al. (2011d)
33	57 year old woman	Her husband	Emmendingen, 2022	Anticoagulants	Anticoagulants could have been phenprocoumon: inhibits the carboxylation and thus the function of the factors II, VII, IX and X, inhibits blood clotting	Case: ZEIT (2022) Phenprocoumon: Seifert (2021i)
34	Two male prisoners (35 and 36 years old)	A fellow inmate	Freiburg prison, 2020	Rat poison	Rat poison is either brodifacoum or thallium Brodifacoum: Inhibits the synthesis of vitamin K-dependent coagulation factors in plasma and liver, inhibits the formation of thrombin and fibrin Thallium: has a toxic effect on the central and peripheral nervous system, liver, kidneys, smooth muscles of the stomach and intestines	Case: ZEIT (2021b) Brodifacoum: Dekant and Vamvakas (2010a) Thallium: Dekant and Vamvakas (2010b)
35	38 year old man	His wife	Nürnberg, 2020	Etizolam	Etizolam is a benzodiazepine: makes receptors more sensitive to GABA (anxiolytic, sedative-hypnotic, muscle relaxant effects), leads to respiratory depression	Case: ZEIT (2021c) Etizolam: Seifert (2021g), Brunton et al. (2011d)
36	unknown	7 persons at the Technical university of Darmstadt	Technical university of Darmstadt, 2021	1,4- Butandiol (BDO), Bromphenol and Dicyclohexylamin	BDO: acts like gamma hydroxy-butyric acid and can cause drowsy states up to coma Dicyclohexylamin: animal experiments showed, for example, damage to the mucous membranes, convulsions, hemorrhages in the lungs, and dyspnea	Case: Bohr and Lehberger (2021) BDO: Irwin (1996) Dicyclohexylamin: BG RCI (2000)
37	Maria Rohrbach (27, female)	Her husband	Münster, 1957	Veronal (sleeping medication)	Veronal is a preparation of Barbital, a barbiturate: modulates the GABA_A_ receptor allosterically, causing hyperpolarisation and inhibition of neuronal activity	Case: Spiegel (1961) Barbiturates: Seifert (2021b), Brunton et al. (2011b)
38	Unknown	Dieter Wagner	Vaterstetten, 1972	E605 (parathion)	E605 is parathion: an organophosphate, inhibits acetylcholinesterase, causes pulmonary oedema, respiratory and cardiac arrest	Case: Spiegel (1972) E605: Dekant and Vamvakas (2010e)
39	Nurse Rudolf Zimmermann (41, male)	9 patients	Oberbarmen, Mettmann, Neviges, Hattingen, Hattingen-Blankenfels, 1971–1973	Opiates, poison and “Betäubungsmittel” (English “narcotics”)	Opiates: MOR agonists, have analgesic, sedative effects, can cause hypotension and bradycardia as well as respiratory depression “Betäubungsmittel” are defined by the German law (“Betäubungsmittelgesetz”) and include substances with a high potential for addiction, for example benzodiazepines, barbiturates and opiates	Case: Spiegel (1974) MOR agonists: Seifert (2021o) Betäubungsmittel: BfArM (2025)
40	Former prostitute Lydia L.-B. (54, female) and her friend Siegmund Sch. (39, male)	4 spouses (Günter Schwenke, Adolf Berge, Paul Gräf, Gerhard Gauger)	Göttingen, around 1994–2000	“Betäubungsmittel” (English “narcotics”)	“Betäubungsmittel” are defined by the German law (“Betäubungsmittelgesetz”) and include substances with a high potential for addiction for example benzodiazepines, barbiturates and opiates	Case: Friedrichsen (2008) Betäubungsmittel: BfArM (2025)
41	Nursing assistant Cornelia S. (50, female)	5 colleagues	Gießen, 2017–2019	Benzodiazepines, Oxazepam	Benzodiazepines: make receptors more sensitive to GABA (anxiolytic, sedative-hypnotic, muscle relaxant effects), lead to respiratory depression Oxazepam is also a benzodiazepine	Case: Eisenhardt (2020) Benzodiazepines: Seifert (2021g), Brunton et al. (2011d)
42	Prison officer (26, male)	1 inmate Wilhelm Gottfried Nowozin	Werl, 1952	Carbon monoxide	Carbon monoxide: binds to hemoglobin, creating carboxyhemoglobin, blocks the oxygen binding site and reduces the oxygen transport capacity	Case: Spiegel (1952) Carbon monoxide: Dekant and Vamvakas (2010f)
43	Sergeant Peter Kellein (25, male)	3 colleagues	Bodelsberg, 1967	Zinc chloride	Zinc chloride: is highly corrosive and can damage the heart, kidneys, blood vessels and the central nervous system	Case: Spiegel (1969) Zinc chloride: Várady (1943)
44	Nurse Niels Högel (23, male)	85 patients	Oldenburg and Delmenhorst, 2000–2018	Gilurytmal	Gilurytmal is a preparation of Ajmaline: a class Ia anti-arrhythmic drug, blocks sodium channels in cardiac muscle cells	Case: Spiegel (2018), Spiegel (2019b), Spiegel (2020b) Gilurytmal: Editorial Gelbe Liste Pharmaindex (2022)
45	Margit Grätz (31, female)	Her husband and her father in law	Munich, 1984–1988	Drain cleaner „Drano-Rohrfrei “	Drain cleaners are caustic alkalis that damage the heart, kidneys, blood vessels and the central nervous system	Case: Friedrichsen (1989) Drain cleaners: Dekant and Vamvakas (2010 g)
46	29 year old woman	Her two children	Immenreuth, 2002	Unknown poison	Not enough information given	Case: Spiegel (2022c)
47	Unknown	2 colleagues	Minden, 2006	Cyanide	Hydrocyanic acid salt: blocks cytochrome oxidase, interrupts the respiratory chain and thus the production of metabolic energy, respiratory paralysis	Case: Spiegel (2006c) Cyanide: Dekant and Vamvakas (2010e)
48	39 year old woman	Her two children	Lünen, 2010	Carbon monoxide	Carbon monoxide: binds to hemoglobin, creating carboxyhemoglobin, blocks the oxygen binding site and reduces the oxygen transport capacity	Case: Spiegel (2010) Carbon monoxide: Dekant and Vamvakas (2010f)
49	32 year old woman	Her son	Schwedt, 1974	Carbon monoxide	Carbon monoxide: binds to hemoglobin, creating carboxyhemoglobin, blocks the oxygen binding site and reduces the oxygen transport capacity	Case: Eisenhardt (2016) Carbon monoxide: Dekant and Vamvakas (2010f)
50	53 year old man	His son	Hamburg, 2017	Unknown poison	Not enough information given	Case: Spiegel (2017)
51	Christel Müller (25, female) and her lover Wilhelm Leinauer (27, male)	Her husband Manfred Müller and his friend Albert Blumoser	Kempten, 1967	Cyanide	Hydrocyanic acid salt: blocks cytochrome oxidase, interrupts the respiratory chain and thus the production of metabolic energy, respiratory paralysis	Case: ZEIT (1967) Cyanide: Dekant and Vamvakas (2010e)
52	47 year old woman	Her father, her aunt and 3 spouses	Krefeld, 1964–1982	E605 (parathion)	E605 is parathion: an organophosphate, inhibits acetylcholinesterase, causes pulmonary oedema, respiratory and cardiac arrest	Case: Gerste (1984) E605: Dekant and Vamvakas (2010e)
53	Christa Lehmann (29, female)	Her husband, her father in law, a friend	Worms, 1952–1954	E605 (parathion)	E605 is parathion: an organophosphate, inhibits acetylcholinesterase, causes pulmonary oedema, respiratory and cardiac arrest	Case: Klee (1995) E605: Dekant and Vamvakas (2010e)
54	Policewoman (40, female) and her colleague (42, female)	Her husband	Reutlingen, 2019	Insulin	Insulin: increase of glucose uptake and glycogen synthesis as well as inhibition of lipolysis, hypoglycaemia	Case: Spiegel (2019c) Insulin: Seifert (2021f), Brunton et al. (2011e)
55	Babysitter (38, female)	2 infants	Berlin, 2019	Methadone	Methadone: a MOR agonist, is used for substitution in MOR agonist addicts	Case: Spiegel (2020c) Methadone: Seifert (2021q)
56	35 year old man	His baby	Potsdam, 2014	Disinfectant	Disinfectant: leads to chemical burns, for example on the esophagus and stomach	Case: Spiegel (2015) Disinfectant: Dekant and Vamvakas (2010 g)
57	Unknown	7 year old Anna B	Tamm-Hohenstange, 1993	Arsenic	Arsenic: capillary toxic effect, causes pain, diarrhea and circulatory shock due to fluid loss	Case: Schrep (2000) Arsenic: Dekant and Vamvakas (2010b)
58	Unknown	40 year old man	Hannover, 2011	Mercury	Mercury: pronounced corrosive effect, can cause bronchitis, bronchiolitis and even pneumonia when inhaled, can cause gastroenteritis with bloody diarrhoea, damages the gastrointestinal tract and kidneys, leads to headaches, dizziness, paralysis, tremors	Case: Spiegel (2012) Mercury: Dekant and Vamvakas (2010e)
59	Miriam P. (39, female)	The spouse of her friend Manfred J	Hof, 2017	Ethylene glycol	Ethylene glycol: leads to metabolic acidosis, kidney failure, can also cause calcium oxalate crystals	Case: Eisenhardt (2018) Ethylene glycol: Ringler et al. (1994)
60	Klaus O. (54, male)	10 colleagues	Schloß Holte-Stukenbrock, 2015–2018	Lead-acetate, cadmium, mercury	Lead: affects the central and peripheral nervous system, haematopoietic system and the kidneys, causing nausea, intestinal colic, constipation, toxic blood count changes, liver and kidney damage, fatigue, headaches and muscle pain Cadmium: causes kidney damage and increased excretion of proteins in the urine, damages tubular cells, interferes with calcium metabolism Mercury: pronounced corrosive effect, can cause bronchitis, bronchiolitis and even pneumonia when inhaled, can cause gastroenteritis with bloody diarrhoea, damages the gastrointestinal tract and kidneys, leads to headaches, dizziness, paralysis, tremors	Case: Parth (2018) Lead-acetate: Dekant and Vamvakas (2010d) Cadmium: Dekant and Vamvakas (2010 h) Mercury: Dekant and Vamvakas (2010e)
61	51 year old woman	Her husband	Berlin, 2017	Thallium	Thallium: has a toxic effect on the central and peripheral nervous system, liver, kidneys, smooth muscles of the stomach and intestines	Case: Spiegel (2021c) Thallium: Dekant and Vamvakas (2010b)
62	30 year old man and his 34 year old girlfriend	His daughter	Hannover, 2023	Mercury	Mercury: pronounced corrosive effect, can cause bronchitis, bronchiolitis and even pneumonia when inhaled, can cause gastroenteritis with bloody diarrhoea, damages the gastrointestinal tract and kidneys, leads to headaches, dizziness, paralysis, tremors	Case: Spiegel (2024a) Mercury: Dekant and Vamvakas (2010e)
63	56 year old man	His wife	Dessau-Roßlau, 2023	Blue monkshood (aconitine)	Blue monkshood (Aconitine): alters the sodium channels of excitable membranes and delays repolarization	Case: Spiegel (2024b) Aconitine: Dekant and Vamvakas (2010c)

To show a change on homicidal poisonings over time, it was decided to split the cases into an older group and a newer group. As a cutoff point the German reunion 1989 was chosen. It was not too long ago and had a big impact on the German society. Many newer developments like the internet also took place after that so that these could have had an impact on the newer cases compared to the older ones. These two groups were analysed all together but also separately to find differences between them. There were only 13 cases before the reunion and 50 after that, so the analysis is more focused on more recent crime cases.

## Results

### Analysis of year and place

**Fig. 2:**
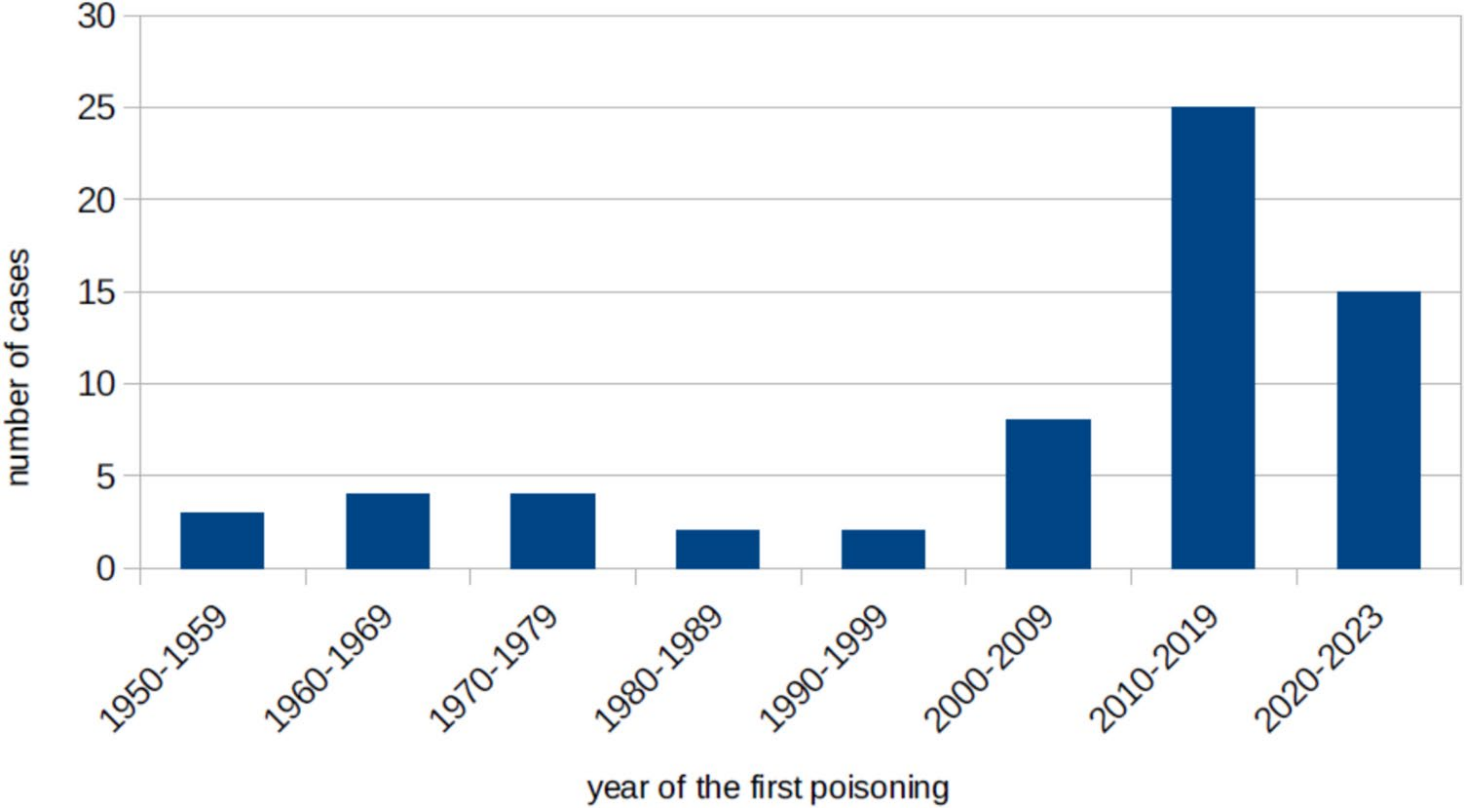
The numbers of cases that were found in each decade, shown in a bar chart

Figure 2 shows the year in which the murder cases took place. If the perpetrator has committed poisonings over several years, the year of the first poisoning was taken. The vast majority (25 cases) came from the years 2010–2019. However, in the years 2020–2023, up to the time of the evaluation, there had already been 15 homicidal poisonings.

**Fig. 3:**
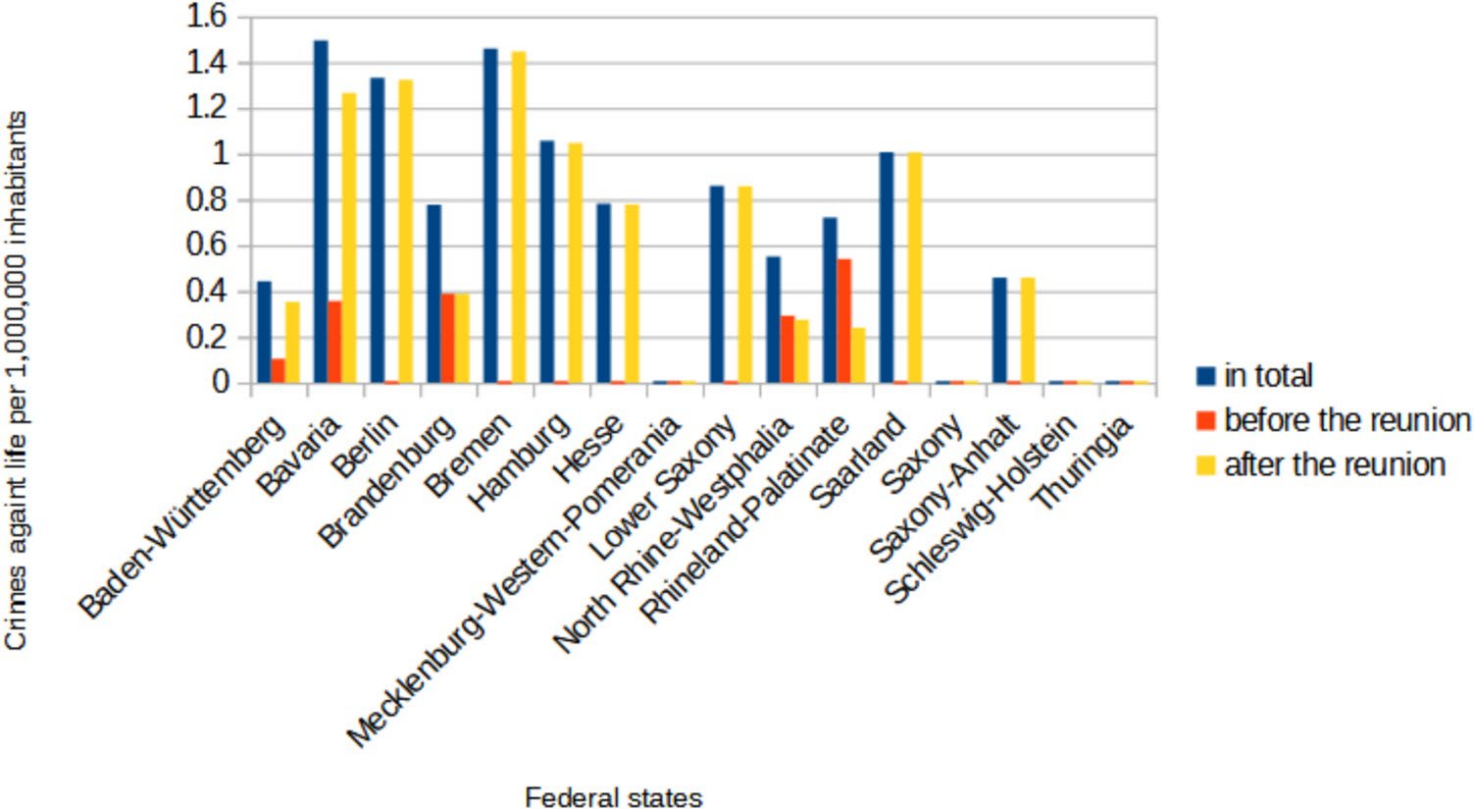
A depiction of where the most crime cases happened. The number of cases in each federal state was calculated per 1,000,000 inhabitants. Shown in a bar chart, where the total value is blue, the value before the reunion is red and the value after the reunion is yellow

On the question of the location (Fig. 3), the cases were assigned to one of the 16 federal states of Germany and calculated per 1,000,000 inhabitants. For the cases after the reunion and the cases in total the population figures of the federal states from 2022 were used (Statistisches Bundesamt (Destatis) 2025a). For the cases before the reunion the population figures from 1989 were used (Statista 2025a; Statista 2025b; Statista 2025c; Statista 2025d; Statistisches Landesamt Baden-Württemberg 2024). In total most cases were found in Bavaria (1.5 cases per 1,000,000 inhabitants), closely followed by Bremen (1.46 cases per 1,000,000 inhabitants) and Berlin (1.3 cases per 1,000,000 inhabitants). No cases were found from Mecklenburg-Western Pomerania, Saxony, Schleswig–Holstein and Thuringia. Before the reunion only a few cases from five federal states (Baden-Württemberg, Bavaria, Brandenburg, North Rhine-Westphalia, Rhineland-Palatinate) were found, after the reunion the cases were spread almost all over Germany.

### Analysis of the poisonings

**Fig. 4:**
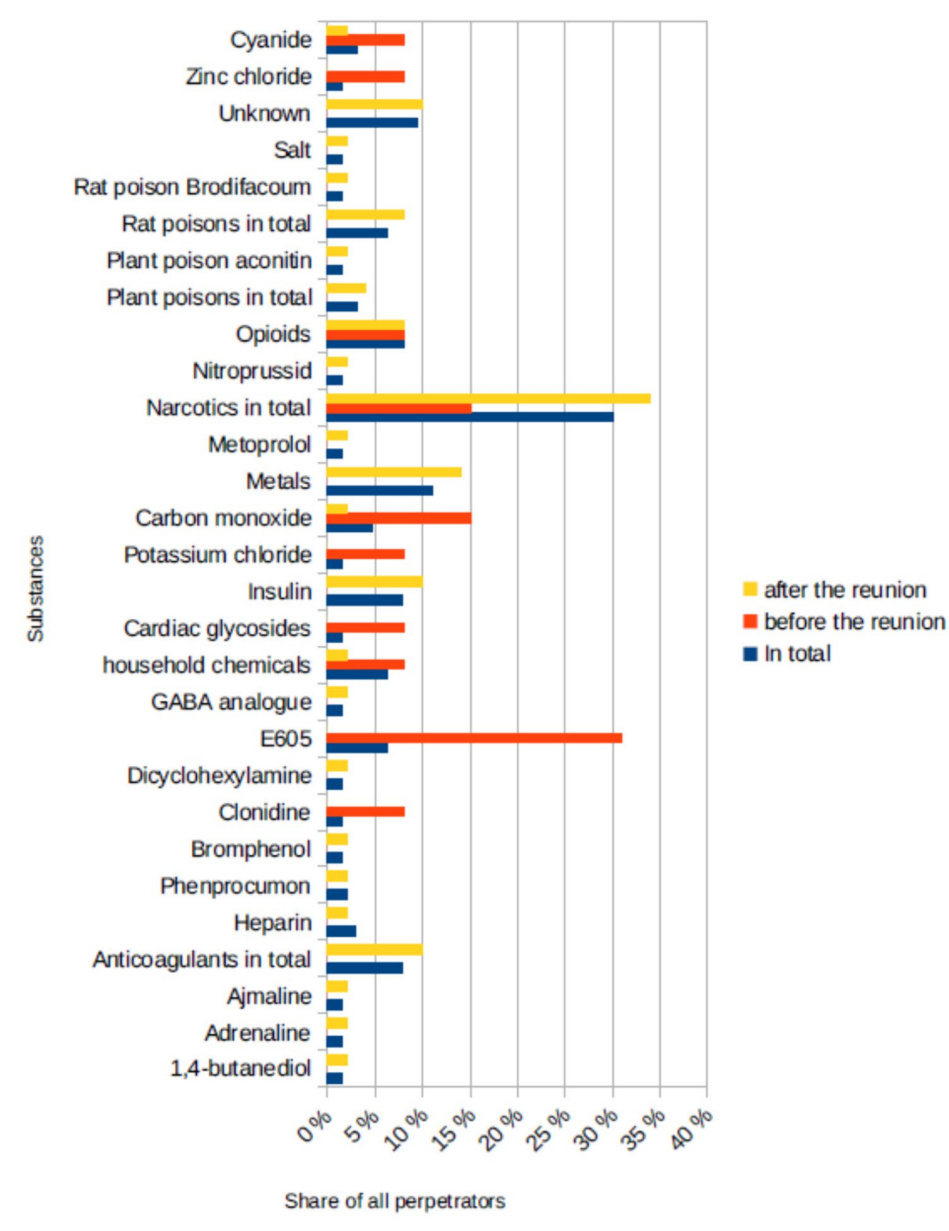
The substances that were used in the poisonings, shown in a bar chart in which the total value (*percentage value of all cases) is blue, the value before the reunion is red and the value after the reunion is yellow

Many different poisons and drugs were used in the criminal cases that were analysed (Fig. 4). The biggest group were narcotics which were used by 30% of all perpetrators (15% before the reunion, 34% after the reunion). Anticoagulants in total (8% of all perpetrators, 0% before the reunion, 10% after the reunion), the insecticide E605, an irreversible acetylcholine esterase inhibitor (6% of all perpetrators, 31% before the reunion, 0% after the reunion), MOR agonists (8% of all perpetrators, 8% before the reunion, 8% after the reunion), insulin (8% of all perpetrators, 0% before the reunion, 10% after the reunion), metals (11% of all perpetrators, 0% before the reunion, 14% after the reunion) and rat poison (6% of all perpetrators, 0% before the reunion, 8% after the reunion) were also used frequently. Before the reunion carbon monoxide was also very common (5% of all perpetrators, 15% before the reunion, 2% after the reunion). In 10% of all cases (0% before the reunion, 10% after the reunion) it is not known which poison the perpetrator used. Some perpetrators used more than one substance.

**Fig. 5:**
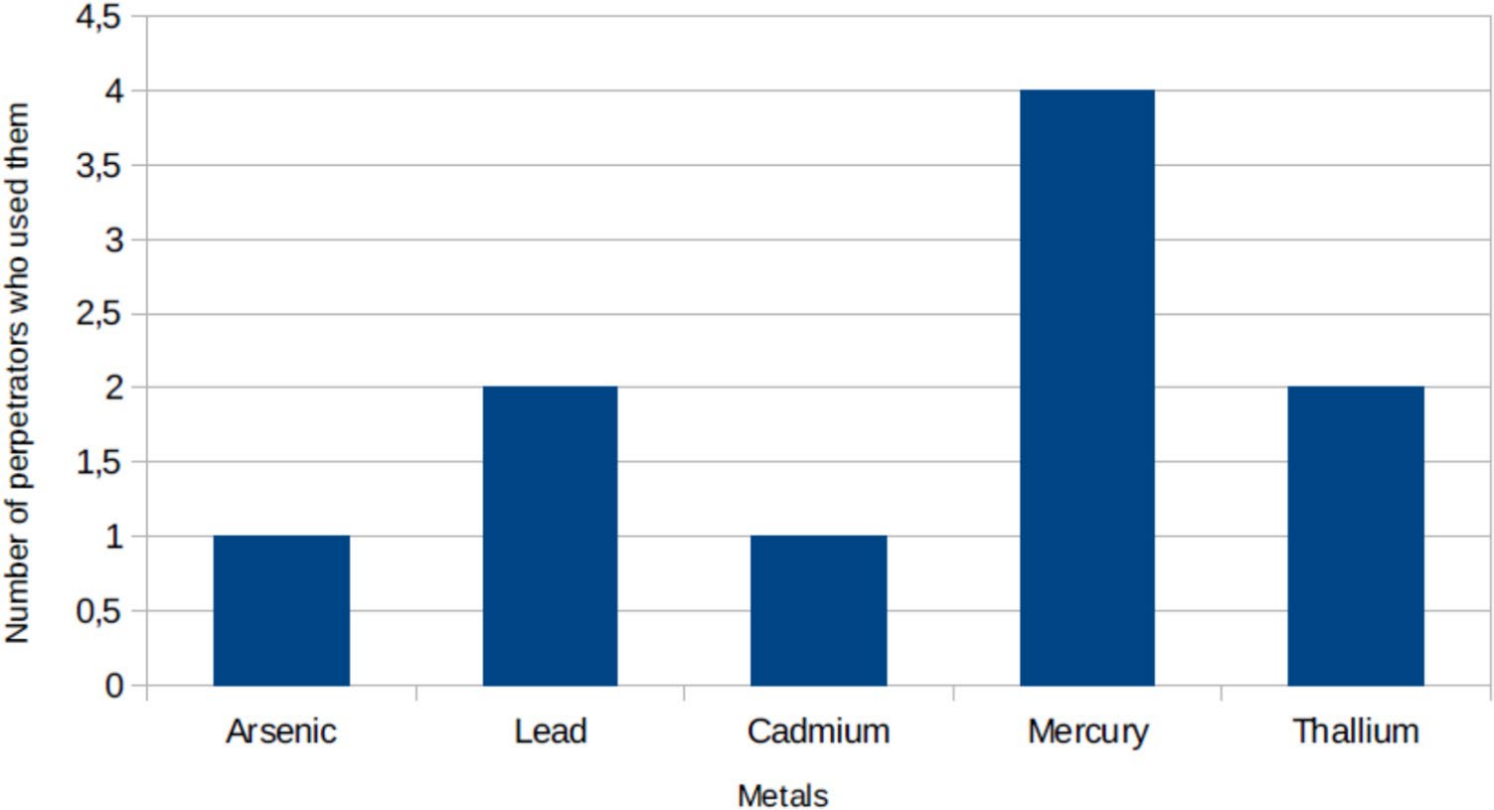
The number of perpetrators that used each metal, shown in a bar chart

In Fig. 5 it is shown in detail which metals were used. Mercury was the used most frequently, by four perpetrators, followed by lead and thallium (each used by three perpetrators).

**Fig. 6:**
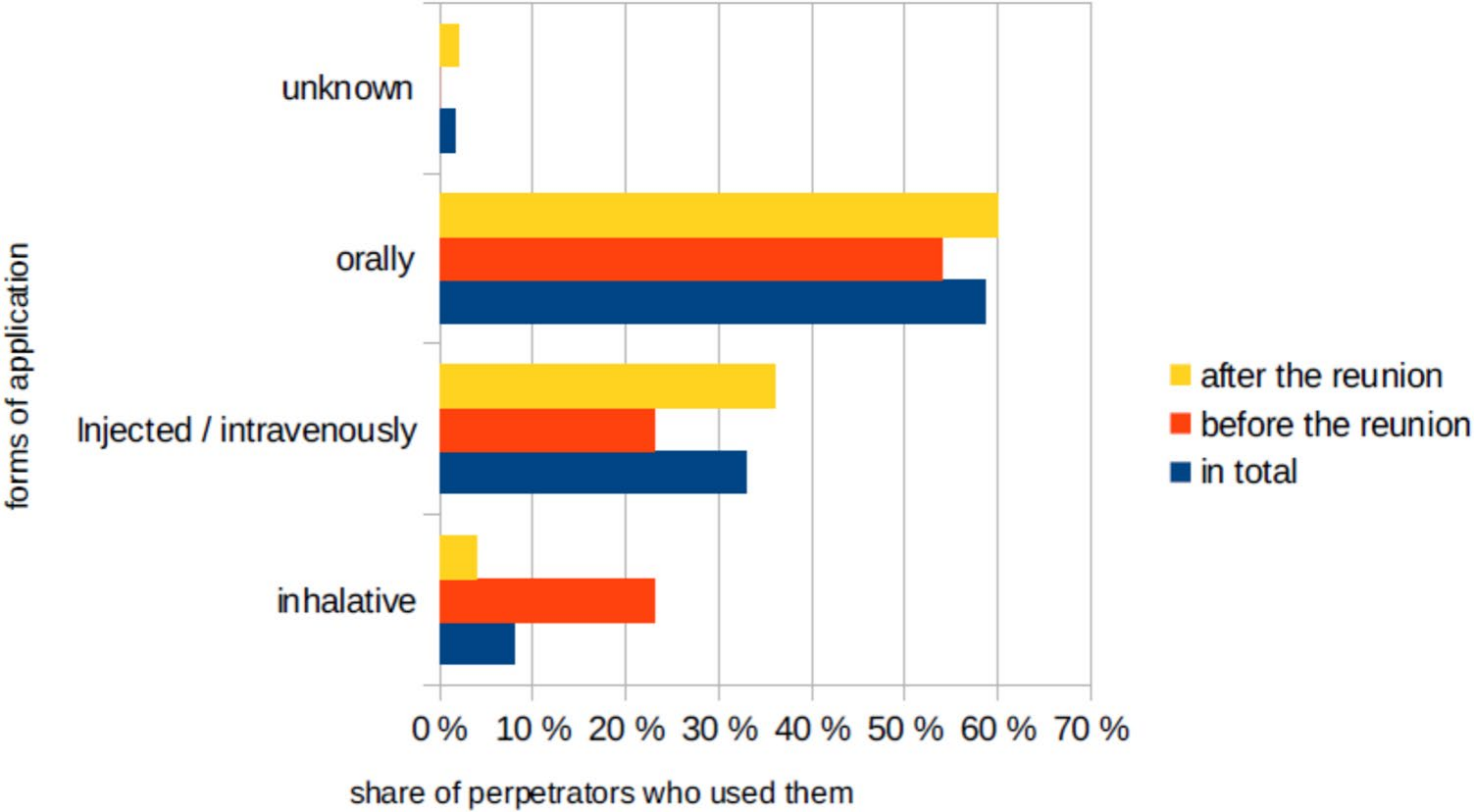
The forms of application shown as the share of perpetrators who used them, shown in a bar chart in which the total value (*percentage value of all cases) is blue, the value before the reunion is red and the value after the reunion is yellow

The most common form of application (Fig. 6) was orally (59% of all perpetrators, 54% before the reunion, 60% after the reunion). Most of the time the poison was put into the food or drink of the victim. The second most common form of application was intravenously or injected (33% of all perpetrators, 23% before the reunion, 36% after the reunion). This form was used mainly by perpetrators in hospitals or other nursing facilities with drugs that are applied this way. Before the reunion inhalation poisonings appeared more frequently (8% of all perpetrators, 23% before the reunion, 4% after the reunion).

**Fig. 7:**
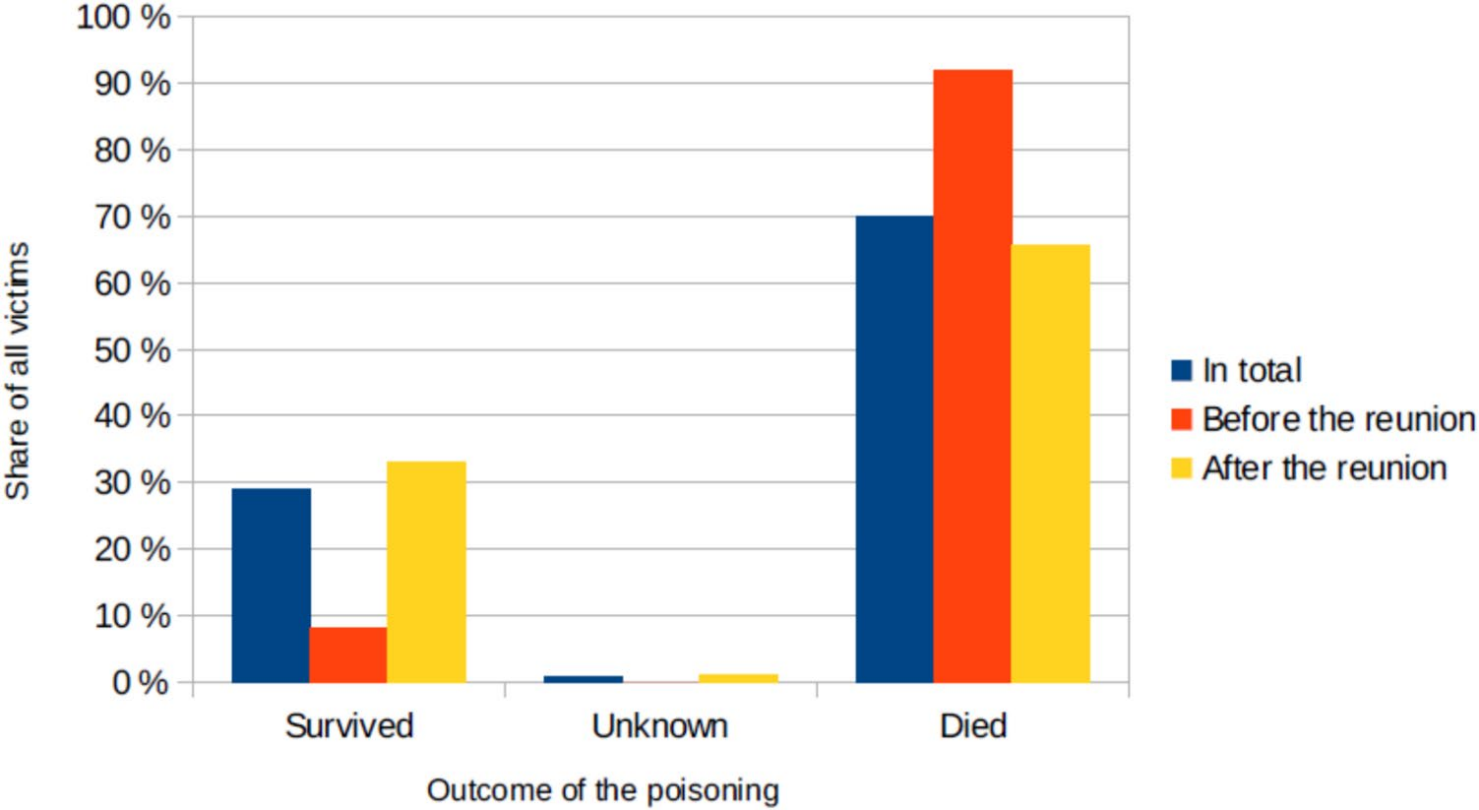
The share of all victims who died or survived the poisoning, shown in a bar chart in which the total value (*percentage value of all cases) is blue, the value before the reunion is red and the value after the reunion is yellow

The outcome (Fig. 7) was fatal in most cases (70% of all victims, 92% before the reunion, 66% after the reunion). On the other hand almost a third survived (29% of all victims, 8% before the reunion, 33% after the reunion). For 1% of the victims the outcome is unknown (0% before the reunion, 1% after the reunion).

**Fig. 8:**
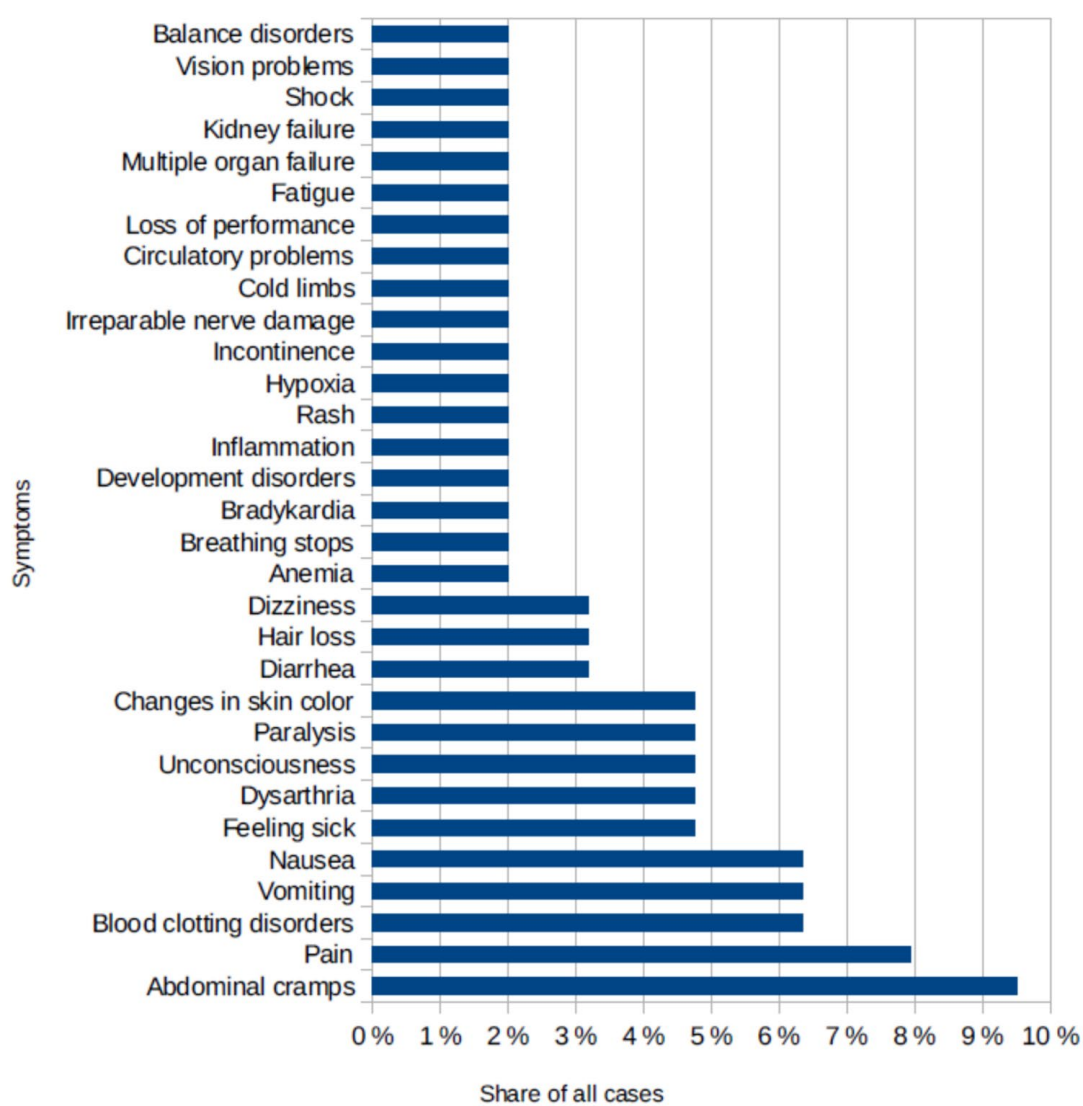
The various symptoms that were mentioned in the newspaper articles, shown in a bar chart in which the total value is the number of articles

When looking at the symptoms described in the articles it becomes clear that poisonings can show a vast variety of symptoms (Fig. 8). There may be abnormalities in various parts of the body such as the gastrointestinal tract, circulatory system, nervous system, skin and others. The symptom that was named the most were abdominal cramps (named in 10% of all cases), followed by some kind of pain (8%), blood clotting disorders (6%), vomiting (6%) and nausea (6%).

### Analysis of the depiction in newspapers

**Fig. 9:**
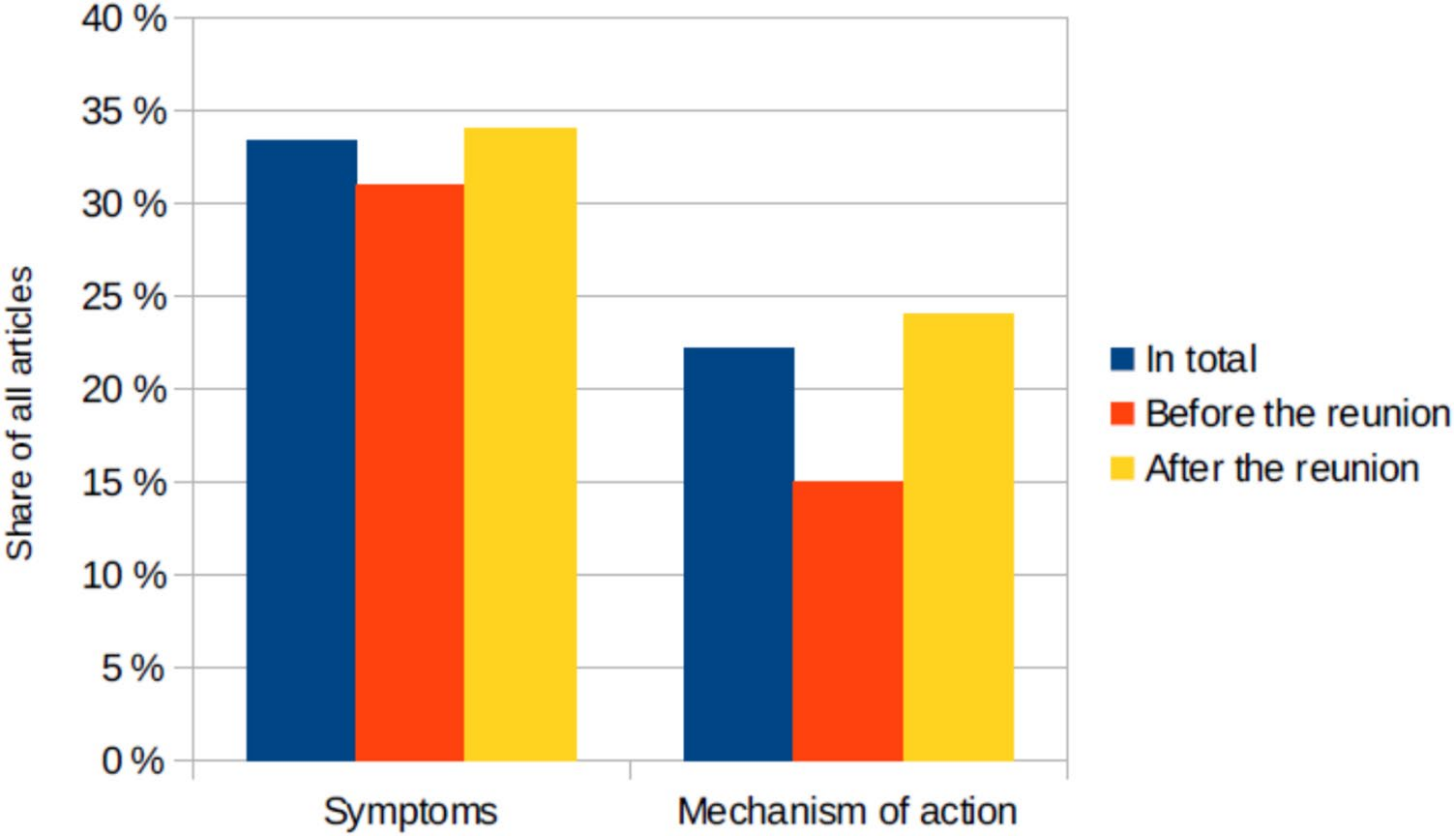
The share of all articles in which symptoms or the mechanism of action were mentioned, shown in a bar chart in which the total value (*percentage value of all articles) is blue, the value before the reunion is red and the value after the reunion is yellow

Although many different symptoms are named throughout the articles, it is important to note that not all articles described the symptoms that occurred. Figure 9 shows that online a third (33% of all articles, 31% before the reunion, 34% after the reunion) of all articles named at least one symptom. Information about the mechanism of action of the drug or poison or in which body part it works is only given in 22% of all articles (15% before the reunion, 24% after the reunion).

**Fig. 10:**
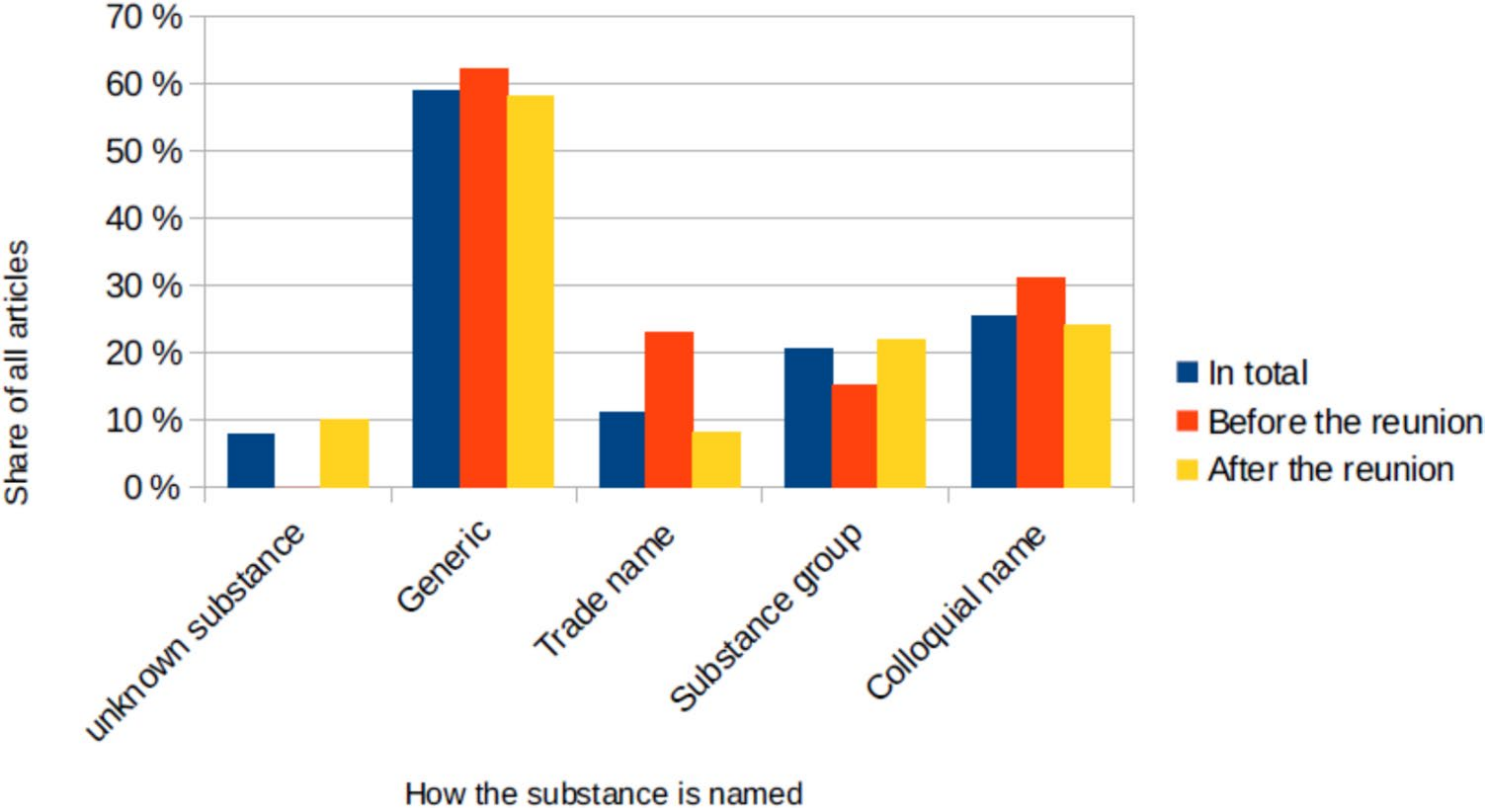
How the substance was named in the newspaper article, shown in a bar chart in which the total value (*percentage value of all articles) is blue, the value before the reunion is red and the value after the reunion is yellow

It is important to look how the substances are called in the article (Fig. 10). Most often the generic name is mentioned (59% of all articles, 62% before the reunion, 58% after the reunion). The trade name (11% of all articles, 23% before the reunion, 8% after the reunion) and colloquial names (25% of all articles, 31% before the reunion, 24% after the reunion) are less common. The substance group (21% of all articles, 15% before the reunion, 22% after the reunion) is most often mentioned when the specific substance is unknown (e.g. sedative, sleeping pills). Many articles used more than one name for the substance.

### Analysis of perpetrator and victim

**Fig. 11:**
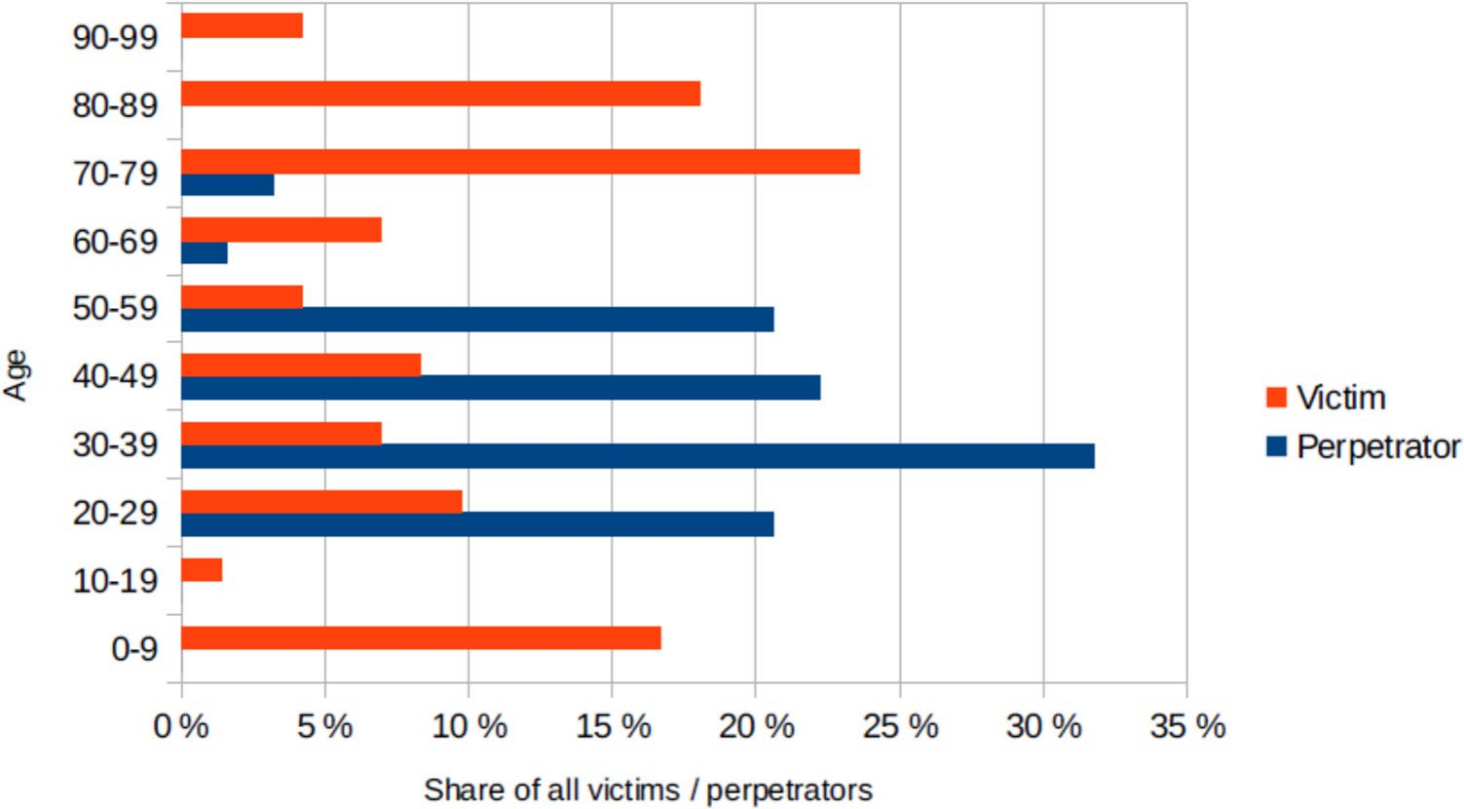
The ages of the perpetrators and victims, shown in a bar chart where the value for victims is red and the value for perpetrators is blue

It stands out that in most crimes the victim is either very old or very young (Fig. 11). 46% of all victims were older than 70 and 17% under 10. The perpetrators are mostly middle aged, the majority was between 30 and 39 (29% of all perpetrators). For better clarity only victims and perpetrators were included whose ages were known.

**Fig. 12:**
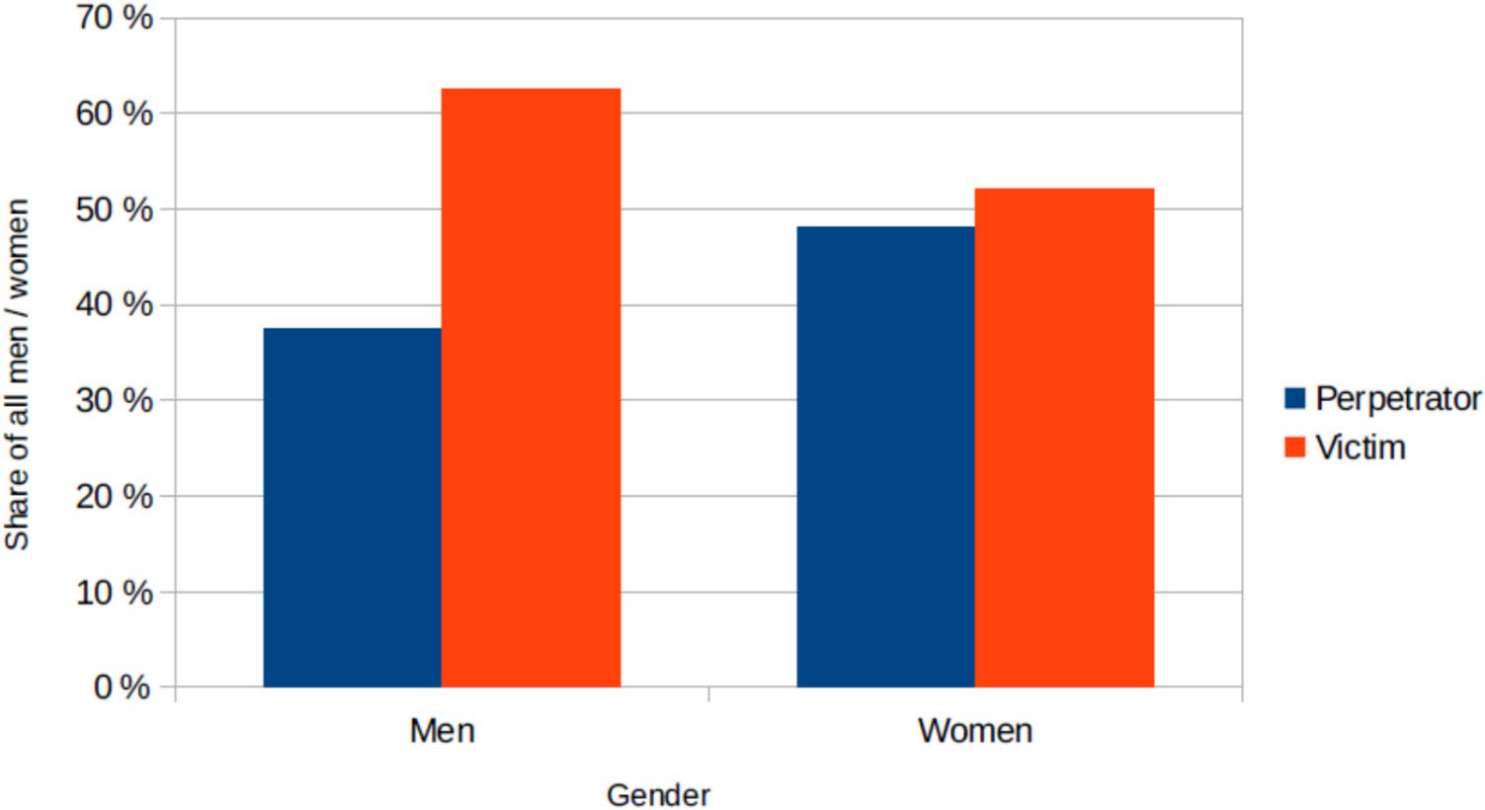
The genders of the perpetrators and victims, shown in a bar chart in which the value for the victims is red and the value for the perpetrators is blue

When analyzing the gender ratios (Fig. 12), it is noticeable that the women involved were perpetrators and victims with approximately equal frequency (48% of women perpetrators, 52% of women victims). Men, on the other hand, are the victims in around two thirds of the cases (62.5% of men victims, 37.5% of men perpetrators).

Figure S2 shows the genders of the perpetrators in detail. Most perpetrators were female in both groups. This is true after the reunion (52% of perpetrators female) but especially before the reunion (57% of perpetrators female, 53% in total).

The genders of the victims on the other hand (Fig. S3) differ before and after the reunion. After the reunion the same share of victims was male and female (50% each). Before the reunion most victims were male (76% of victims). Therefore, the victims overall tend to be male (57%).

Figure S4 shows how many victims the perpetrators poisoned or wanted to do so. About half of all perpetrators only had one victim (46% of all perpetrators, 38% before the reunion, 48% after the reunion), the other half more than one. 25% had 3–10 victims (38% before the reunion, 22% after the reunion) and 10% more than 10 (8% before the reunion, 10% after the reunion).

The identity of the perpetrator (Fig. S5) is most often the life partner or former life partner of the victim (35% of all perpetrators, 46% before the reunion, 32% after the reunion). In second place the perpetrator was part of the medical staff that treated the victim as a patient (25% of all perpetrators, 23% before the reunion, 26% after the reunion). It is also common for the perpetrator to be a parent (13% of all perpetrators, 8% before the reunion, 14% after the reunion) or child (8% of all perpetrators, 15% before the reunion, 6% after the reunion). In 10% (8% before the reunion, 10% after the reunion) of the cases the perpetrator was not found or was unknown to the victim.

### Investigation and background of the cases

Fig. S6 shows where or how the perpetrator got the poison they used. In 17% of the cases (23% before the reunion, 16% after the reunion), the poison or drug came from the pharmacy of the hospital where the perpetrator worked. 16% of perpetrators (38% before the reunion, 10% after the reunion) purchased and used an over-the-counter or everyday substance and 10% (0% before the reunion, 12% after the reunion) purchased the substance online, some of them internationally. 5% of the perpetrators (8% before the reunion, 4% after the reunion) received a substance from other people who, for example, take this medicine themselves or had other access to the substance. In 46% of cases (15% before the reunion, 54% after the reunion) it is not known where the poison came from or it was not mentioned in the article.

The most frequently mentioned motive (Fig. S7) was interpersonal problems (27% of all perpetrators, 23% before the reunion, 28% after the reunion). Then comes greed (14% of all perpetrators, 8% before the reunion, 16% after the reunion) and stress or being overwhelmed (11% of all perpetrators, 15% before the reunion, 10% after the reunion). In many cases the motive is unknown (29% of all perpetrators, 38% before the reunion, 26% after the reunion) and often there were multiple factors and different reasons that lead to the poisoning which are still not easy to grasp.

Fig. S8 shows by whom the poisoning was noticed or how the idea that the victim might have been poisoned first arose. In 25% of the cases (23% before the reunion, 26% after the reunion) there is no information about this. In most cases, the poisoning was suspected by the victims treating doctor (17% of all cases, 8% before the reunion, 20% after the reunion) or by the victims environment (14% of all cases, 0% before the reunion, 20% after the reunion), much more often than by the victim himself (2% of all cases, 8% before the reunion, 0% after the reunion). In 10% of the cases (23% before the reunion, 6% after the reunion) hospital staff suspected a colleague because of accumulating deaths for example and in 6% of the cases (0% before the reunion, 8% after the reunion) the victim noticed something with their senses like a strange taste in their food.

In some of the cases analyzed, court rulings were described (Fig. S9). The most common punishment was life sentence (14% of perpetrators, 15% before the reunion, 14% after the reunion). The second most common were prison sentences between 5 and 10 years (11% of perpetrators, 0% before the reunion, 12% after the reunion). A preventive detention was ordered in 2% of all cases (0% before the reunion, 2% after the reunion) and 5% of perpetrators (0% before the reunion, 6% after the reunion) received a professional ban.

## Discussion

### Differences between accidental and homicidal poisonings

Poisonings with malicious or even lethal intentions are very rare. This emerges from a publication by the “Bundesgesundheitsblatt” based on previous publications (Hahn et al. 2014). According to this, there were 207,000 poisonings in Germany between 2005 and 2012, which shows that it is not a rare phenomenon. The results from 2011 were broken down in more detail. Poisonings caused by external influences were 0%, accidents (67%) and suicides (20%) were much more common. This is also reflected in this analysis, which found only 63 cases of homicidal poisonings since the 1950s. Since in the analysis of the “Bundesgesundheitsblatt” a poisoning was defined as one person who got poisoned, the numbers of this analysis were converted and adjusted accordingly.

A big difference between overall poisonings and homicidal poisonings is the choice of substance. Homicidal poisonings were largely caused by medication (72% of victims). In the data from the “Bundesgesundheitsblatt”, medicinal products were also the largest group, but the proportion was significantly lower (39%). On the other hand, the proportion of chemical and physicochemical agents such as cleaning agents was significantly larger (26%) than in homicidal poisonings (1%), as was the proportion of plants (10% in the “Bundesgesundheitsblatt”, 1% in homicidal poisonings).

An important difference also lies in the outcome of the poisoning. While 70% of the victims of homicides died, poisonings that are largely accidental in origin are very rarely fatal (less than 0.1%) and serious injuries are also rare (2%). But probably, attempted homicidal poisonings without serious outcomes rarely come to light.

A third important difference is the age distribution of the victims. In the data from the “Bundesgesundheitsblatt”, most of the victims were small children, most often between 1 and 2 years old. There is a second peak in the age distribution among young adults, among whom suicidal poisoning accounts for the largest proportion. Children were also a large group in the homicidal poisonings (17% of victims under 10 years of age), but the largest proportion were seniors over 70 (46% of victims).

### Homicide-suicides

Among the collected cases, there were two cases of homicide-suicide. In the case number 48, a mother killed herself and her two children (Spiegel 2010) and in case number 50 a father poisoned himself and his son (Spiegel 2017). It is a rare phenomenon in Germany, the perpetrators of homicide-suicide are usually men who commit the act with firearms (Hellen et al. 2014). The victim is usually the partner, but also often the children. Women more often take the children with them in their deaths. Many perpetrators suffer from depression or other mental illnesses. According to psychiatrist Prof. Dr. Ulrich Hegerl, the killing of the children could be motivated by a sense of responsibility, because people do not want to leave the children behind (Schlee 2006).

### Analysis of the year and place of the poisoning

When analyzing the years (Fig. 2) of homicidal poisonings, there was a sharp increase in cases after 2010. Data from the “Bundeskriminalamt” (Federal Criminal Police Office, BKA) could help (Birger 2017). According to this, murders and manslaughters rose to 1,468 cases per year by 1993 and fell again to 565 by 2015. This does not match the data found in the archive of *Der Spiegel* and other newspapers. The theory that more murders involving poisons and drugs were reported in the 2010 s seems more logical. The development of the Internet and better accessibility to information could argue for this. *Der Spiegel* published an average of 211 articles per week online in 2023 (Radü 2023). There weren’t even half as many articles in the printed magazines of the 1980 s as can be seen in the online archive. With so many more articles, it seems logical that more attention could be paid to the topic of homicidal poisonings, since there is no limited space online.

When looking at the federal states in which the crimes happened (Fig. 3), there were no murders in the federal states of Mecklenburg-Western Pomerania, Thuringia, Saxony and Schleswig–Holstein. Statistics from the BKA were also used for this purpose (Bundeskriminalamt 2025a). Fig. S10 shows the crimes against life in 2023 per 1,000,000 inhabitants in each federal state. Here a similar trend is seen, fewer crimes tended to be committed in the federal states of Mecklenburg-Western Pomerania, Saxony and Thuringia. As can be seen in Fig. 11, older people are also less likely to be perpetrators and the federal states of Mecklenburg-Western Pomerania, Thuringia, Saxony and Schleswig–Holstein are among the states with the highest average age, while the states with the most poisonings (Bavaria, Berlin and Bremen) are among the federal states with the lowest average age (Statista 2025e). In addition, only 63 cases were evaluated in this analysis, so some random fluctuation is expected.

### Analysis of the poisonings

Figure 4 shows that a large proportion of the crimes were committed with narcotics. This is not surprising considering that 25% of the perpetrators were medical staff who have easy access to this kind of medication. The fact that the accessibility of a substance plays a major role in the choice can be clearly seen from the insecticide E605. The organophosphate used to be easily accessible. In 1954 it gained great attention as a means of killing through the case of Christa Lehmann, who is considered the first murderer with E605 (case number 53). Between 1952 and 1954 she killed three people after learning of its toxicity. The case received a lot of media attention and made many people aware of the substance. It became a popular suicide method and it is believed that hundreds of thousands of people took their own lives using it (Wahrig 2025). In this analysis, the last murder case involving E605 was in 1982. E605 was banned in 2002.

Instead, poisonings involving metals have become more common. Heavy metal poisoning is also a common problem as an accident and has been reported more frequently in the recent past. The “Verbraucherzentrale” (“consumer advice agency”) warns of high concentrations of heavy metals, for example in products containing seaweed, mineral clay or ayurvedic products (Verbraucherzentrale 2024). We have recently reviewed more than 200 cases of lead and arsenic intoxication by traditional and alternative medicine (Gerke and Seifert 2025). Given the knowledge of the toxic effects of heavy metals, it is not surprising that perpetrators also resort to them more often.

In recent articles it is often not known which substance was used for the poisoning. While this fact was always mentioned in old cases, it was completely unknown in 10% of newer cases. One reason for this could be the increase in articles and cases, which means that newspapers are less restricted in their choice of topics and report on cases where little information is available. In the age of the Internet, the faster availability of information might also play a role and it is possible that an event is reported quickly by newspapers without the case being completely solved.

The most common form of application was oral (Fig. 6), which seems logical as this is the least invasive. It probably takes less effort for a perpetrator to mix a poison into a victim’s food or drink than to kill the victim violently. The second most common form of application, intravenously or injected, is just as logical, as most perpetrators were medical staff and most patients on wards have an intravenous access that could be used.

The symptoms mentioned in the articles (Fig. 8) are very diverse and show the readers how diverse a poisoning can manifest itself. This is good for raising public awareness of poisonings and possibly detecting the problem earlier.

### Analysis of the depiction in newspapers

The fact that the newspapers show a wide range of possible symptoms of poisoning is diminished by the fact that only 34% of the articles mention at least one symptom (Fig. 9). Here, newspapers miss a great opportunity to educate and enlighten their readers. If the general population knew more about symptoms of poisoning in the future, they would be able to identify symptoms in themselves or other people more quickly, which could help raise awareness and, in the best-case scenario, save lives. Information about the mechanism of action is also only provided in 23% of the articles. These would further contribute to the readers’ understanding and knowledge. Overall, better education in these aspects would be helpful to prevent and detect poisonings in the future.

When looking at the depiction of the poisonings in the newspapers, it is important to consider how they name the substances (Fig. 10). What is positive here is that the generic or scientific name is mentioned in many articles. In some articles only the trade name was mentioned. This could give the impression that only this preparation is dangerous, and laypeople do not realize that there are other preparations with the same active ingredient that could have the same effect. However, the additional mention of a trade name or a colloquial term can arouse associations in the reader that the generic name would not have triggered because they only know the trade names The mention of several names and an explanation of these could inform and educate the reader pharmacologically about drugs that he may be familiar with and whose danger he was not aware of. It would therefore be best if more pharmacological information were provided in articles, but of course it should be scientifically correct and thus the use of the generic name should be promoted.

### Analysis of perpetrator and victim

Perpetrators are mostly middle-aged (Fig. 11). At this age they are the strongest physically and overall, the most independent, which is in stark contrast to the victims. Most victims are very old or very young. This could be explained by the fact that these are particularly vulnerable groups who cannot defend themselves so easily and are in special need of protection. However, there were no cases of Münchhausen-by-proxy-syndrome in this analysis. The only gap in the ages of perpetrators and victims is the 10–19-year-olds. It is very likely that there are also some young perpetrators who are simply not reported in the media. In Germany, the main hearings for criminals under the age of 18, unlike adults, do not take place in public. In addition, special personal protection must be provided to protect perpetrators from stigmatization (Die Justiz des Landes NRW 2025). It is therefore reasonable to assume that perpetrators in this age group were not reported publicly.

As shown in Figure S2, the perpetrators in this analysis were predominantly female. The proportion of women among poisoners is significantly higher than among violent crimes overall. Statistics from the BKA from 2024 show that the proportion of women among murderers in 2023 was 14%, and among manslaughters it was only 11% (Bundeskriminalamt 2025b). People need less physical strength and violence to commit a poisoning (Neuen-Biesold 2011). The fact that women were historically more involved in cooking and nursing could also have contributed to this. In addition, female violence is most often directed against one’s own partner, for example to free oneself from violent relationships. Women typically kill after a long period of suffering rather than in a moment of passion. It seems logical that when a crime is planned, one would rather choose poison as the method of murder, as it leaves less obvious traces such as blood or injuries and could rather be mistaken for a natural death.

According to an analysis by the Criminal Investigation Department (Die Kriminalpolizei 2010), violence by female perpetrators is most often directed against their partner or their own children, which aligns with Figure S5, because the perpetrators in this analysis were most often a partner or former partner of the victim.

It is also fitting that the victims are more often male (Fig. S3), because according to statistics from the BKA, the proportion of women among murder victims in 2023 was 41%, and the proportion among homicide victims was only 27% (Bundeskriminalamt 2025c).

The fact that the proportion of male victims was even higher in the years before the reunion is consistent with the fact that the perpetrators were also more often partners of the victim before the reunion. The women’s rights movement may have played an important role. Since it used to be very difficult for women to divorce from their husbands and lead independent lives, they may have chosen the method of murder. For example, improvements in working conditions for women, better contraceptive options, changes in marriage and divorce laws and improved pension rights could have played a role (Universität Bielefeld 2025).

If a person commits murder, there is a high probability that he will continue to murder (Fig. 10). The reason for this is probably a lowered inhibition threshold. Anyone who has successfully murdered once without getting caught likely has less inhibitions about doing it a second time. This can be clearly seen, for example, in the case of Christa Lehmann (case 53). After successfully murdering her husband, she did it again on her father-in-law and tried it a third time on a friend’s mother when a new problem occurred.

However, it is also true that a large proportion of serial killers did not decide to kill again only after the first crime. According to the Criminal Investigation Department (Die Kriminalpolizei 2010), there are fundamental differences between one-time perpetrators and serial perpetrators. The big difference is probably that although both try to solve their problem with the poisoning, only the one-time perpetrators succeed. If a woman kills her abusive husband, for example, her problem is gone. However, if people kill because they want to exercise power over other people or because they are overwhelmed by their work in an intensive care unit (Fig. S7), then the act does not solve their basic problem or the need is only satisfied for a short time, which is why it is likely that they will strike again.

The fact that some perpetrators murdered more than ten people before they were discovered can be explained by the fact that many perpetrators murdered in hospitals, sometimes even in intensive care units. For old and sometimes seriously ill people, death often does not come as a surprise, which is probably why the circumstances were not investigated in more detail and the cases were only discovered because of other causes.

This analysis found that in most cases the perpetrator was one’s own partner or former partner (Fig. S5). According to the BKA, this is also the case in 23% of murders and 29% of manslaughters (Bundeskriminalamt 2025b). According to their statistics, in 34% of murders and 25% of manslaughters, perpetrators and victims are unrelated. In this analysis, this was rather rare, which could have something to do with the fact that poisonings, as already described, often follow a long, painful period and therefore have a specific cause. Just to kill, perpetrators often choose other methods that may be easier to implement and require less planning.

An exception to this could be hospitalized offenders who have easier access to medication. According to an article by the Criminal Investigation Department, the number of perpetrators belonging to medical personnel is increasing worldwide (Die Kriminalpolizei 2010). One reason for this could be an insecure personality of the perpetrators, which together with professional and private conflicts leads to an overload situation. Accordingly, many people go into the health sector who have little self-love and many insecurities because they hope for recognition and gratitude from this fulfilling profession. However, this is often not the case in the health sector. Many patients are very old and there is no improvement in their health. There are many chronic illnesses and everyday working life is filled with stress. Especially in intensive care units, very rationalized, mechanical work often dominates compared to work in which one receives positive feedback from people. Perpetrators then project their own suffering onto the people they are supposed to be helping and kill them in the hope of ending their own pain.

While responsibility obviously lies with the perpetrators, circumstances can also trigger an act. Constantly dealing with people in need of care, sick people and death can be a psychologically stressful situation for many people, which not everyone can handle well. The fact that stress and feeling overwhelmed can be reasons for violent crimes was also shown in the motives mentioned in the newspaper articles (Fig. S7). A study by the “Deutsches Ärzteblatt” (German Medical Journal) on 70 serial murders in medical facilities also revealed that several circumstances often coincide, such as a high workload, serious illnesses of the patient, a difficult working environment, and great responsibility in the job (Dettmeyer et al. 2023).

There is great potential here to prevent murders in the future. Due to demographic change and an increasingly ageing society, the need for care will become a major problem. According to the Federal Statistical Office, the number of people over 85 in Germany will almost double by 2055 (Statistisches Bundesamt (Destatis) 2025b).

This requires relief for caring relatives and medical staff to prevent stress and overloads and thus violent crime in the future. More time for patients, a stronger focus on people instead of profits, better working conditions and more recognition for the work of medical staff are important steps to take.

### Investigation and background of the cases

When it came to the origin of the poison (Fig. S6), the most common information given was that the perpetrator got the poison from the hospital where they worked. The poison was taken from the ward or ordered through the hospital pharmacy. A security gap in hospitals is noticeable here, as more drugs were ordered and consumed in the specific wards than was ordered by the doctor. In the future, murders could be prevented with better security measures and more attention from staff.

The serial killer Niels Högel has already triggered first corresponding developments. He was convicted in 85 cases in 2019. As a result of the case, the Lower Saxony state parliament passed a new hospital law. By 2022, the clinics were supposed to set up ward pharmacists and drug commissions to monitor the dispensing of drugs more closely (DAZ-Redaktion 2019). Furthermore, anonymous reporting points are being set up where one can report criminal behaviour or abnormalities. Conferences will be held to investigate deaths and suspicious incidents.

In its investigation into serial murders in hospitals, the “Deutsches Ärzteblatt” (German Medical Journal) has also published a long list of suspicious factors that warrant attention (Dettmeyer et al. 2023). These include an excessive frequency of deaths, the unexplained discovery of syringes, irregularities in medication inventory, and much more. It is also recommended to enable anonymous reporting, offer training, and actively work against looking away.

It is also important to note that in many articles it is not explained how the perpetrator got the substance they used. This is especially true for cases that happened after the reunion. The main reason is probably that this information should not be public to not help future perpetrators.

The motives mentioned for the crimes (Fig. S7) were varied. Often several factors came together and, as already described, the personality of the perpetrator certainly plays a major role. The motives nevertheless provide important clues as to how future crimes can be prevented, for example by relieving the burden on medical staff or creating more psychological support services. Other studies have also found certain character traits that are more common in serial killers (Dettmeyer et al. 2023). They often have narcissistic ideas of power and superiority, a strong desire for recognition, and dehumanize their victims.

The poisoning was most often suspected by a doctor treating the victim (Fig. S8), significantly more often than by the victim himself. Doctors are in a crucial position here. Of course, medical laypeople should get better education and information to classify their symptoms themselves, which could be improved through newspapers, for example, but in the end doctors are the ones who are turned to when symptoms are unclear. It is therefore important that especially those who are the first point of contact, such as family doctors or medical staff in the emergency room and emergency services, think of poisonings and consider crimes if the symptoms are unclear.

Even after death, it is important to discover and solve the crimes. The autopsy also played an important role in these cases. In 10% of cases, the autopsy revealed the poisoning. However, an autopsy is not carried out for every death and not every poisoning is visible in an autopsy. One has to look for many substances specifically and these would not be detected in a routine autopsy without the suspicion of a poisoning (Kinast 2019). In this aspect there is a lot of room for improvement in the future by enhancing the methods for detecting poisons during autopsies and, ideally, searching for them more frequently. This is of course a cost–benefit question, but cheaper methods and better options could help detect more poisonings in the future.

In the few cases in which a verdict was mentioned ranged from suspended sentences to three life sentences or sentences of particularly serious guilt (Fig. S9). This wide range of judgments can be explained by the importance of the exact circumstances. Whether there was an intent to kill, how many victims there were, the motive, the exact method, the amount of poison, previous convictions and whether the perpetrator showed remorse could all have had an influence on each specific case.

### Research gap

As already mentioned, there is little data on homicidal poisonings in Germany. This may be since it is a rare phenomenon. Only 63 cases were found in this study. For example, there were only seven cases of metal poisoning. Ethical principles and moral concepts may also argue against researching real cases, to protect the privacy and data protection of the victims. As already mentioned, there are also large numbers of unreported cases, as not every poisoning is detected and is often difficult to prove (Kinast 2019). Court cases are also imperfect as sources of scientific evidence, as witnesses, for example, have subjective perceptions and memories, and courts adhere to formal standards and don’t necessarily seek the truth. All these factors could discourage researchers from conducting more research into homicidal poisonings.

## Limitations

A major limitation is that only poisonings that were reported in the four newspapers were analyzed, which presumably means that mostly media effective cases were included. Since a search function was used, it cannot be ruled out that there were cases in the newspapers that were not found using the specific search terms. This resulted in fewer cases than initially hoped, namely 63. Especially before the German reunion, there were only 13 cases, which means that statistical statements can only be made to a limited extent. The information on the individual cases also came from newspapers, which may have contained errors. Often not all aspects of the crime were known. The poisons and drugs mentioned were often only named by the substance group they belong to, which made it difficult to understand which substance it was based on the information given. In addition, not all cases were fully resolved at the time of the research and many still had court proceedings pending. In addition, only cases from Germany were considered, from which one can only draw national conclusions. There is also little information about poisonings in Germany and this relates almost exclusively to accidents, which meant that it was hardly possible to compare the data collected to existing one or to put it into context.

## Conclusions and future studies

This analysis has shown that homicides involving poisons occur in Germany. In the private sector, it is a popular method of getting rid of one’s partner and there have been several serial killers in German hospitals who have murdered dozens of patients. The victims are often either children or older people in need of care who are killed by perpetrators who want to exercise power or who are overwhelmed by the work in care. The availability of substances always played a major role, as can be seen looking at the drug E605 or at medical staff who ordered the killing drugs from the hospital pharmacy. Security gaps continue to emerge here, which have already been responded to with a new hospital law to better detect the misuse of drugs. Serial perpetrators often have complex problems that are difficult to address. Unfortunately, the newspapers that report on the cases often miss their potential to enlighten and educate the reader. Instead of just reporting on the dramaturgy of the criminal case, they could convey more pharmacological content such as information about the exact substance or the symptoms that occur in the event of a poisoning. Better education for readers could help identify poisonings more quickly and treat them better in the future.

Much further research is needed in this field. Suicides involving poisons and drugs were not analysed here and differences between male and female perpetrators were only touched upon. Since this analysis only analyses newspapers, the question arises as to how homicidal poisonings are portrayed in other media such as podcasts, videos or the news. Homicidal poisonings in fictional media also represent a large field of research in which one could examine how realistic they are and how the depictions affect society. Finally, data from other countries could reveal interesting differences from which important conclusions could be drawn about preventing homicidal poisonings in the future.

## Supplementary Information

Below is the link to the electronic supplementary material.

ESM1 (DOCX 1.29 MB)

## Data Availability

All original data for this study are available from the authors upon reasonable request.

## Abbreviations

BKA: Bundeskriminalamt (Federal Criminal Police Office)
GABA: Gamma-aminobutyric acid
MOR: μ-Opioid receptor
SZ: Süddeutsche Zeitung

## References

[CR1] AOK (2022) Giftige Pflanzen im Garten: Gefahr für Kind und Tier, Das AOK-Gesundheitsmagazin https://www.aok.de/pk/magazin/familie/eltern/10-giftige-pflanzen-im-heimischen-garten/ Accessed 21 Aug 2025

[CR2] Arndt C, Wulf H (2016) Hypernatremia – diagnostics and therapy. Anasthesiol Intensivmed Notfallmed Schmerzther 51:308–315. 10.1055/s-0041-107265 27213601 10.1055/s-0041-107265

[CR3] Bernstein M (2018) 73-Jährige soll Giftmord an Ehemann versucht haben, Süddeutsche Zeitung, https://www.sueddeutsche.de/muenchen/neuperlach-73-jaehrige-soll-giftmord-an-ehemann-versucht-haben-1.4015935 Accessed 08 Jan 2025

[CR4] BfArM (2025) Betäubungsmittel https://www.bfarm.de/DE/Bundesopiumstelle/Betaeubungsmittel/_node.html Accessed 14 Jan 2025

[CR5] BG RCI (2000) Toxikologische Bewertungen, edn. 11/00, Heidelberg, pp 9ff

[CR6] Birger A (2017) Morde 1950 bis 2015, Die Kriminalpolizei, Verlag deutscher Polizeiliteratur https://www.kriminalpolizei.de/ausgaben/2017/maerz/detailansicht-maerz/artikel/morde-1950-bis-2015.html Accessed 14 January 2025

[CR7] Brunton L, Chabner B, Knollman B (2011a) Specific parenteral agents. In: Goodman & Gilman’s The Pharmacological Basis of Therapeutics, Twelfth Edition, The McGraw-Hill Companies, Inc., p 536

[CR8] Brunton L, Chabner B, Knollman B (2011b) Specific parenteral agents. In: Goodman & Gilman’s The Pharmacological Basis of Therapeutics, Twelfth Edition, The McGraw-Hill Companies, Inc., p 533

[CR9] Brunton L, Chabner B, Knollman B (2011c) Parenteral anticoagulants. In: Goodman & Gilman’s The Pharmacological Basis of Therapeutics, Twelfth Edition, The McGraw-Hill Companies, Inc., pp 853ff

[CR10] Brunton L, Chabner B, Knollman B (2011d) Anesthetic adjuncts. In: Goodman & Gilman’s The Pharmacological Basis of Therapeutics, Twelfth Edition, The McGraw-Hill Companies, Inc., p 548

[CR11] Brunton L, Chabner B, Knollman B (2011e) Insulin Therapy. In: Goodman & Gilman’s The Pharmacological Basis of Therapeutics, Twelfth Edition, The McGraw-Hill Companies, Inc., p 1248

[CR12] Bohr F, Lehberger R (2021) LKA Hessen findet Hinweise auf Giftanschlag mit K.o.-Tropfen, Spiegel, https://www.spiegel.de/panorama/justiz/tu-darmstadt-lka-hessen-findet-hinweise-auf-giftanschlag-mit-k-o-tropfen-a-6ca248db-918a-49d4-9771-eb16b79ff1c6 Accessed 10 January 2025

[CR13] Bundeskriminalamt (2024) Häusliche Gewalt im Jahr 2023 um 6,5 Prozent gestiegen, https://www.bka.de/DE/Presse/Listenseite_Pressemitteilungen/2024/Presse2024/240607_PM_BLB_Haeusliche_Gewalt.html#:~:text=Ganz%20%C3%BCberwiegend%20trifft%20Gewalt%20im,etwa%20aus%20Angst%20oder%20Scham. Accessed 21 August 2025

[CR14] Bundeskriminalamt (2025a) T01 Grundtabelle - Fälle -Länder (V1.0) https://www.bka.de/DE/AktuelleInformationen/StatistikenLagebilder/PolizeilicheKriminalstatistik/PKS2023/PKSTabellen/LandFalltabellen/landFalltabellen.html?nn=226064 Accessed 14 January 2025

[CR15] Bundeskriminalamt (2025b) T20 Tatverdächtige insgesamt nach Alter und Geschlecht (V1.0) https://www.bka.de/DE/AktuelleInformationen/StatistikenLagebilder/PolizeilicheKriminalstatistik/PKS2023/PKSTabellen/BundTV/bundTV.html?nn=226082 Accessed 15 January 2025

[CR16] Bundeskriminalamt (2025c) T91 Opfer insgesamt nach Alter und Geschlecht (V1.0) https://www.bka.de/DE/AktuelleInformationen/StatistikenLagebilder/PolizeilicheKriminalstatistik/PKS2023/PKSTabellen/BundOpfertabellen/bundopfertabellen.html?nn=226082 Accessed 15 January 2025

[CR17] DAZ-Redaktion (2019) Nach Klinikmorden: Einführung von Stationsapothekern und Meldestellen, Deutsche Apotheker Zeitung, https://www.deutsche-apotheker-zeitung.de/daz-az/2019/daz-24-2019/nach-klinikmorden-einfuehrung-von-stationsapothekern-und-meldestellen Accessed 15 January 2025

[CR18] Dekant W, Vamvakas S (2010a) Pestizide. Toxikologie: Eine Einführung für Chemiker, Biologen und Pharmazeuten, 2nd edn. Spektrum Akademischer Verlag, Heidelberg, p 214

[CR19] Dekant W, Vamvakas S (2010b) Schwermetalle und Metalloide. Toxikologie: Eine Einführung für Chemiker, Biologen und Pharmazeuten, 2nd edn. Spektrum Akademischer Verlag, Heidelberg, p 189

[CR20] Dekant W, Vamvakas S (2010c) Pflanzengifte. Toxikologie: Eine Einführung für Chemiker, Biologen und Pharmazeuten, 2nd edn. Spektrum Akademischer Verlag, Heidelberg, pp 248f

[CR21] Dekant W, Vamvakas S (2010d) Schwermetalle und Metalloide. Toxikologie: Eine Einführung für Chemiker, Biologen und Pharmazeuten, 2nd edn. Spektrum Akademischer Verlag, Heidelberg, pp 183f

[CR22] Dekant W, Vamvakas S (2010e) Umweltschadstoffe. Toxikologie: Eine Einführung für Chemiker, Biologen und Pharmazeuten, 2nd edn. Spektrum Akademischer Verlag, Heidelberg, pp 177ff

[CR23] Dekant W, Vamvakas S (2010f) Umweltschadstoffe. Toxikologie: Eine Einführung für Chemiker, Biologen und Pharmazeuten, 2nd edn. Spektrum Akademischer Verlag, Heidelberg, p 176

[CR24] Dekant W, Vamvakas S (2010g) Akute Vergiftungen. Toxikologie: Eine Einführung für Chemiker, Biologen und Pharmazeuten, 2nd edn. Spektrum Akademischer Verlag, Heidelberg, p 27

[CR25] Dekant W, Vamvakas S (2010h) Schwermetalle und Metalloide. Toxikologie: Eine Einführung für Chemiker, Biologen und Pharmazeuten, 2nd edn. Spektrum Akademischer Verlag, Heidelberg, p 182

[CR26] Dettmeyer R, Saß H, Malolepszy L, Mousa M, Teske J, Vennemann B (2023) Serial killings and attempted serial killings in hospitals, nursing homes, and nursing care. Dtsch Arztebl Int 120:526–533. 10.3238/arztebl.m2023.0128 37278091 10.3238/arztebl.m2023.0128 PMC10534130

[CR27] Die Justiz des Landes NRW (2025) Das Jugendstrafverfahren, Justiz NRW, https://www.justiz.nrw/BS/lebenslagen/Strafrecht/BesondereVerfahrensarten/jugendstrafverfahren Accessed 15 January 2025

[CR28] Die Kriminalpolizei (2010) Wenn Frauen töten, Verlag deutscher Polizeiliteratur, https://www.kriminalpolizei.de/ausgaben/2010/september/detailansicht-september/artikel/wenn-frauen-toeten.html Accessed 15 January 2025

[CR29] Dimsdale, J. E. (2024). Auf andere übertragene artifizielle Störung (Münchhausen-by-proxy-Syndrom, University of California, San Diego, https://www.msdmanuals.com/de/heim/psychische-gesundheitsst%C3%B6rungen/somatische-belastungsst%C3%B6rung-somatic-symptom-disorder-ssd-und-verwandte-st%C3%B6rungen/auf-andere-%C3%BCbertragene-artifizielle-st%C3%B6rung Accessed 21 August 2025

[CR30] Dionet, R (2007) Der Tod kam durch die Infusion, STERN, https://www.stern.de/panorama/verbrechen/der-giftmord-von-koenigsbrunn-der-tod-kam-durch-die-infusion-3222034.html Accessed 04 January 2025

[CR31] Editorial Gelbe Liste Pharmindex (2022) Ajmalin. Gelbe Liste. Pharmindex. https://www.gelbe-liste.de/wirkstoffe/Ajmalin_1318 Accessed 3 January 2025

[CR32] Eisenhardt U (2015) Mordanschlag aus Angst vor dem Verlassenwerden, STERN, https://www.stern.de/panorama/verbrechen/blei--und-quecksilbervergiftung--ehemann-bringt-lieber-seine-frau-um--als-von-ihr-verlassen-zu-werden-6342530.html Accessed 04 January 2025

[CR33] Eisenhardt U (2016) Erna F. und ihr toter Junge im Kinderbett, Spiegel, https://www.spiegel.de/panorama/justiz/mordprozess-nach-42-jahren-erna-f-und-ihr-toter-junge-im-kinderbett-a-1115171.html Accessed 10 January 2025

[CR34] Eisenhardt U (2018) Vergiftete Beziehung, Spiegel, https://www.spiegel.de/panorama/justiz/hof-frau-wegen-giftmordes-vor-gericht-a-1219848.html Accessed 10 January 2025

[CR35] Eisenhardt U (2020) Gift in der Frühschicht, Spiegel, https://www.spiegel.de/panorama/justiz/giessen-prozess-gegen-pflegehelferin-gift-in-der-fruehschicht-a-58e0489e-91ce-4150-aab4-5048e618deed Accessed 10 January 2025

[CR36] Friedrichsen G (1989) „Daß endlich amal a Ruh’ werd“, Spiegel, https://www.spiegel.de/politik/dass-endlich-amal-a-ruh-werd-a-a4f2b68a-0002-0001-0000-000013493650 Accessed 10 January 2025

[CR37] Friedrichsen G (2008) „Die alten Männer wollten immer grapschen bei ihr“, Spiegel, https://www.spiegel.de/panorama/justiz/prozess-gegen-schwarze-witwe-die-alten-maenner-wollten-immer-grapschen-bei-ihr-a-536328.html Accessed 10 January 2025

[CR38] Gerke L, Seifert R (2025) Lead and arsenic intoxications by traditional and alternative medicine: men are more sensitive than women. Naunyn Schmiedebergs Arch Pharmacol 398:799–818 39066909 10.1007/s00210-024-03317-y PMC11787186

[CR39] Gerste M (1984) Sie wollte nur Ruhe, ZEIT, https://www.zeit.de/1984/40/sie-wollte-nur-ruhe Accessed 10 January 2025

[CR40] Hahn A, Begemann K, Stürer A (2014) Vergiftungen in Deutschland. Krankheitsbegriff, Dokumentation und Einblicke in das Geschehen [Cases of poisoning in Germany. Disease entity, documentation, and aspects of the event]. Bundesgesundheitsbl Gesundheitsforsch Gesundheitsschutz 57(6):638–649. 10.1007/s00103-014-1965-9 10.1007/s00103-014-1965-9 24863706

[CR41] Hellen F, Lange-Asschenfeldt C, Huckenbeck W, Hartung B (2014) Der “erweiterte Suizid”. Vollendete Homizid-Suizide unter psychopathologischen und kriminologischen Aspekten ["Extended suicide". Homicide-suicide under psychopathological and criminological aspects]. Nervenarzt 85(9):1144–50. 10.1007/s00115-013-3942-1 24441846 10.1007/s00115-013-3942-1

[CR42] Hinneburg I (2015) Strophanthin – Hilfe für das Herz? https://medizin-transparent.at/strophanthin-das-verschwundene-herzmedikament/ Accessed 3 January 2025

[CR43] Iijima S (2020) Suicide attempt using potassium tablets for congenital chloride diarrhea: A case report. World J Clin Cases. Apr 26;8(8):1463-1470. doi: 10.12998/wjcc.v8.i8.1463. PMID: 32368538; PMCID: PMC7190950. 10.12998/wjcc.v8.i8.1463 PMC7190950 32368538

[CR44] Irwin RD (1996) NTP summary report on the metabolism, disposition, and toxicity of 1,4-butanediol (CAS No. 110–63–4). Toxic Rep Ser. (54):1–28, A1–8, B1–5. PMID: 11803699. 11803699

[CR45] Jüttner J (2010) „Ich bereue nichts“, Spiegel, https://www.spiegel.de/panorama/justiz/todesengel-aus-der-charite-ich-bereue-nichts-a-688151.html Accessed 10 January 2025

[CR46] Kinast J (2019) Toxikologe: „Viele Giftmorde bleiben wohl unentdeckt“, Westdeutsche Zeitung, https://www.wz.de/panorama/toxikologe-thomas-daldrup-viele-giftmorde-bleiben-wohl-unentdeckt_aid-47396193 Accessed 15 January 2025

[CR47] Klee E (1995) Die Geschichte der Giftmörderin, ZEIT, https://www.zeit.de/1995/36/Die_Geschichte_der_Giftmoerderin Accessed 10 January 2025

[CR48] Lakotta B (2021) Frau Dr. Dr. med. Tod, Spiegel, https://www.spiegel.de/panorama/justiz/mordprozess-gegen-falsche-aerztin-von-fritzlar-fuenf-patienten-tot-a-f2166e93-a3b7-487a-975f-b1682cfa81c3 Accessed 10 January 2025

[CR49] Mauz G (1989) „… wir haben es mit eiskaltem Mord zu tun“, Spiegel, https://www.spiegel.de/politik/wir-haben-es-mit-eiskaltem-mord-zu-tun-a-99dd360d-0002-0001-0000-000013493778 Accessed 10 January 2025

[CR50] Meedia (2025) True crime ist beliebteste Podcast-Kategorie, Johann Oberauer GmbH, https://meedia.de/news/beitrag/19233-true-crime-ist-beliebteste-podcast-kategorie.html, Accessed 20 August 2025

[CR51] Neuen-Biesold A (2011) Interview mit Dr. Erika Eikermann, Die PTA in der Apotheke, https://www.diepta.de/news/interview-mit-dr-erika-eikermann Accessed 15 January 2015

[CR52] Neumann C (2004) „Er wollte Seelen befreien“, Spiegel, https://www.spiegel.de/politik/er-wollte-seelen-befreien-a-11bcfdf5-0002-0001-0000-000034076860 Accessed 10 January 2025

[CR53] Parth C (2018) Quecksilber auf dem Pausenbrot, Spiegel, https://www.spiegel.de/panorama/justiz/bielefeld-der-mann-der-seine-arbeitskollegen-vergiftet-haben-soll-a-1238613.html Accessed 10 January 2025

[CR54] Podius (2025) Podcast Charts des Jahres 2024, PODIUS.io GmbH, https://www.podius.io/podcast-charts-of-the-year-2024/germany/all-genres Accessed 20 August 2025

[CR55] Radü J (2023) Wie wir Geld verdienen – und Sie uns lesen, Spiegel, https://www.spiegel.de/backstage/fuenf-jahre-spiegel-in-der-analyse-ueber-wen-schreiben-wir-am-meisten-was-wird-am-besten-gelesen-a-11e8f9e6-0ab0-4b74-8996-1b6677cc9b15 Accessed 14 January 2025

[CR56] Ramm W (2019) „Danach ging ich schlafen“, Spiegel, https://www.spiegel.de/panorama/justiz/prozess-gegen-pflegehelfer-grzegorz-w-und-die-frage-nach-dem-mord-motiv-a-1298611.html Accessed 10 January 2025

[CR57] Ramm W (2020) „Man kann das Atmen vergessen“, Spiegel, https://www.spiegel.de/panorama/justiz/regensburg-toxikologe-in-mordprozess-man-kann-das-atmen-vergessen-a-f41d6209-5e4a-44f2-a4d8-ec31b0c42436 Accessed 10 January 2025

[CR58] Ramm W (2024) Totschlag in bester Absicht, Spiegel, https://www.spiegel.de/panorama/justiz/berlin-urteil-gegen-oberarzt-der-charite-totschlag-in-bester-absicht-a-4397cd03-fa11-47b6-9d57-6b92fc162df2 Accessed 10 January 2025

[CR59] Ringler S, Gmuer R, Faber K, Bleisch J, Müggler SA (1994) CME: Ethylenglykol-Intoxikation [CME: Ethylene Glycol Intoxication]. Praxis Bern 107(20):1097–1106. 10.1024/1661-8157/a003071 10.1024/1661-8157/a003071 30278847

[CR60] Schlee A (2006) Interviewpartner zum Thema erweiterter Suizid, Informationsdienst Wissenschaft e. V., https://idw-online.de/de/news180738 Accessed 20 August 2025

[CR61] Schober, W. (2025). Giftgefahren im Haus, Bayerisches Landesamt für Gesundheit und Lebensmittelsicherheit, Bayrisches Staatsministerium für Umwelt- und Verbraucherschutz https://www.vis.bayern.de/produkte_energie/giftgefahren/gift_im_haus.htm#:~:text=stark%20variieren):-,Reinigungs%2D%2C%20Wasch%2D%20und%20Putzmittel,%C3%9Cbelkeit%2C%20Erbrechen%20und%20Durchfall%20f%C3%BChren. Accessed 21 August 2025

[CR62] Schrep B (2000) Der ungesühnte Tod der Anna B., Spiegel, https://www.spiegel.de/panorama/der-ungesuehnte-tod-der-anna-b-a-5db007a4-0002-0001-0000-000015985974 Accessed 10 January 2025

[CR63] Seifert R (2021a) Prinzipien der Inhalationsnarkose. In: Basiswissen Pharmakologie, 2nd edn. Springer, Berlin, p 379

[CR64] Seifert R (2021b) Wichtige Arzneistoffe zur Durchführung einer Injektionsnarkose. In: Basiswissen Pharmakologie, 2nd edn. Springer, Berlin, p 381

[CR65] Seifert R (2021c) Pharmakologie des cholinergen und adrenergen Systems. In: Basiswissen Pharmakologie, 2nd edn. Springer, Berlin, p 90

[CR66] Seifert R (2021d) Pathophysiologie thromboembolischer Erkrankungen und pharmakologische Eingriffsmöglichkeiten. In: Basiswissen Pharmakologie, 2nd edn. Springer, Berlin, p 279

[CR67] Seifert R (2021e) Wichtige klinische Studien zur Pharmakotherapie der Herzinsuffizienz. In: Basiswissen Pharmakologie, 2nd edn. Springer, Berlin, p 258

[CR68] Seifert R (2021f) Insuline. In: Basiswissen Pharmakologie, 2nd edn. Springer, Berlin, pp 291ff

[CR69] Seifert R (2021g) Pathophysiologie und Pharmakotherapie der koronaren Herzerkrankung. In: Basiswissen Pharmakologie, 2nd edn. Springer, Berlin, p 363

[CR70] Seifert R (2021h) Pharmakologie des cholinergen und adrenergen Systems. In: Basiswissen Pharmakologie, 2nd edn. Springer, Berlin, p 93

[CR71] Seifert R (2021i) Pathophysiologie thromboembolischer Erkrankungen und pharmakologische Eingriffsmöglichkeiten. In: Basiswissen Pharmakologie, 2nd edn. Springer, Berlin, p 280

[CR72] Seifert R (2021j) Pharmakologie des NO-cGMP-Systems. In: Basiswissen Pharmakologie, 2nd edn. Springer, Berlin, p 140

[CR73] Seifert R (2021k) Prinzipien der Inhalationsnarkose. In: Basiswissen Pharmakologie, 2nd edn. Springer, Berlin, p 378

[CR74] Seifert R (2021l) Acetylcholinrezeptoren und Adrenozeptoren. In: Basiswissen Pharmakologie, 2nd edn. Springer, Berlin, p 95

[CR75] Seifert R (2021m) Pharmakologie des cholinergen und adrenergen Systems. In: Basiswissen Pharmakologie, 2nd edn. Springer, Berlin, p 94

[CR76] Seifert R (2021n) Pharmakologische Ansatzpunkte zur Schmerztherapie. In: Basiswissen Pharmakologie, 2nd edn. Springer, Berlin, p 151

[CR77] Seifert R (2021o) Pharmakologische Ansatzpunkte zur Schmerztherapie. In: Basiswissen Pharmakologie, 2nd edn. Springer, Berlin, p 152

[CR78] Seifert R (2021p) Wichtige Arzneistoffe zur Behandlung von Epilepsien und anderen neuropsychiatrischen Erkrankungen mit einem neuronalen Ungleichgewicht. In: Basiswissen Pharmakologie, 2nd edn. Springer, Berlin, p 361

[CR79] Seifert R (2021q) MOR-Agonisten. In: Basiswissen Pharmakologie, 2nd edn. Springer, Berlin, p 161

[CR80] Spiegel (1952) Der Brief des Joneleit https://www.spiegel.de/politik/der-brief-des-joneleit-a-cb29d3ed-0002-0001-0000-000021976692 Accessed 10 January 2025

[CR81] Spiegel (1961) Suchten und fanden https://www.spiegel.de/politik/suchten-und-fanden-a-2001bb35-0002-0001-0000-000043364733 Accessed 10 January 2025

[CR82] Spiegel (1969) „DER MANN HATTE ANGST“ https://www.spiegel.de/politik/der-mann-hatte-angst-a-810a1daf-0002-0001-0000-000045861230 Accessed 10 January 2025

[CR83] Spiegel (1970) Schmeckt so komisch https://www.spiegel.de/politik/schmeckt-so-komisch-a-3661c03e-0002-0001-0000-000045197349 Accessed 10 January 2025

[CR84] Spiegel (1972) Aus der Ferne https://www.spiegel.de/politik/aus-der-ferne-a-7058190b-0002-0001-0000-000042928468 Accessed 10 January 2025

[CR85] Spiegel (1974) Unterm Leichentuch https://www.spiegel.de/politik/unterm-leichentuch-a-55f9f0b2-0002-0001-0000-000041722014 Accessed 10 January 2025

[CR86] Spiegel (1976) Was im Muskel https://www.spiegel.de/politik/was-im-muskel-a-65ca897c-0002-0001-0000-000041330665 Accessed 10 January 2025

[CR87] Spiegel (2006a) Stiefmutter muss nicht ins Gefängnis https://www.spiegel.de/panorama/justiz/toedlicher-pudding-stiefmutter-muss-nicht-ins-gefaengnis-a-406352.html Accessed 10 January 2025

[CR88] Spiegel (2006b) Ermittler gehen von Mord aus https://www.spiegel.de/panorama/justiz/getoetete-charite-patienten-ermittler-gehen-von-mord-aus-a-441652.html Accessed 10 January 2025

[CR89] Spiegel (2006c) Kollege des toten BASF-Mannes sollte ebenfalls vergiftet werden https://www.spiegel.de/panorama/justiz/toedliche-limo-kollege-des-toten-basf-mannes-sollte-ebenfalls-vergiftet-werden-a-455659.html Accessed 10 January 2025

[CR90] Spiegel (2010) Spaziergänger finden Frauenleiche https://www.spiegel.de/panorama/justiz/familiendrama-in-nrw-spaziergaenger-finden-frauenleiche-a-685233.html Accessed 10 January 2025

[CR91] Spiegel (2012) 40-Jähriger stirbt nach rätselhafter Spritzenattacke https://www.spiegel.de/panorama/justiz/quecksilbervergiftung-mann-stirbt-nach-angrif-mit-spritze-in-hannover-a-832677.html Accessed 10 January 2025

[CR92] Spiegel (2013) 25 Menschen essen Brötchen mit Rattengift https://www.spiegel.de/panorama/niedersachsen-25-menschen-essen-broetchen-mit-rattengift-a-894866.html Accessed 10 January 2025

[CR93] Spiegel (2015) Vater soll Kind monatelang Gift verabreicht haben https://www.spiegel.de/panorama/justiz/prozess-in-potsdam-vater-soll-kind-gift-verabreicht-haben-a-1021575.html Accessed 10 January 2025

[CR94] Spiegel (2017) Vater soll Sohn und sich selbst vergiftet haben https://www.spiegel.de/panorama/justiz/familiendrama-in-hamburg-vater-soll-sohn-und-sich-selbst-vergiftet-haben-a-1139829.html Accessed 10 January 2025

[CR95] Spiegel (2018) Der Serienmörder von der Intensivstation https://www.spiegel.de/panorama/justiz/niels-hoegel-prozess-in-oldenburg-der-serienmoerder-von-der-intensivstation-a-1234931.html Accessed 10 January 2025

[CR96] Spiegel (2019a) Mordversuch auf Frühchenstation – lebenslange Haft für Krankenschwester https://www.spiegel.de/panorama/justiz/marburg-mordversuch-auf-fruehchenstation-lebenslange-haft-fuer-krankenschwester-a-1298769.html Accessed 10 January 2025

[CR97] Spiegel (2019b) Ermittler erhebt Vorwürfe gegen Krankenhaus https://www.spiegel.de/panorama/justiz/niels-hoegel-ermittler-erhebt-vorwuerfe-gegen-krankenhaus-delmenhorst-a-1246262.html Accessed 10 January 2025

[CR98] Spiegel (2019c) Polizistin gesteht Anschlag auf Ehemann https://www.spiegel.de/panorama/justiz/tuebingen-polizistin-gesteht-anschlag-auf-ehemann-a-1295627.html Accessed 10 January 2025

[CR99] Spiegel (2019d) Ehefrau zu zehneinhalb Jahren Haft verurteilt https://www.spiegel.de/panorama/justiz/muenchen-gift-im-kartoffelsalat-74-jaehrige-zu-haftstrafe-verurteilt-a-1268916.html Accessed 10 January 2025

[CR100] Spiegel (2020a) Nudelsuppe vergiftet – Täter zu lebenslanger Haft verurteilt https://www.spiegel.de/panorama/justiz/wiesbaden-nudelsuppe-vergiftet-taeter-zu-lebenslanger-haft-verurteilt-a-cf62cfb1-7fa4-4aaf-b3fe-9ead4d2e354e Accessed 10 January 2025

[CR101] Spiegel (2020b) Bundesgerichtshof bestätigt Urteil gegen Niels Högel https://www.spiegel.de/panorama/justiz/niels-hoegel-bundesgerichtshof-bestaetigt-urteil-gegen-patientenmoerder-a-4b751fdc-82d5-410d-b94d-14e603f5fde2 Accessed 10 January 2025

[CR102] Spiegel (2020c) Babysitterin verabreicht Säugling Methadon – sechseinhalb Jahre Haft https://www.spiegel.de/panorama/justiz/babysitterin-verabreicht-saeugling-methadon-sechseinhalb-jahre-haft-a-3cee3009-cf64-4c26-9ef8-65af7eb06d40 Accessed 10 January 2025

[CR103] Spiegel (2021a) 41-Jähriger soll Frauen mit Schwermetall vergiftet haben – zwei Tote https://www.spiegel.de/panorama/justiz/huerth-41-jaehriger-soll-frauen-mit-schwermetall-vergiftet-haben-zwei-tote-a-77fc9826-b688-43ef-be11-f3db60d081e4 Accessed 10 January 2025

[CR104] Spiegel (2021b) Lange Haft für Mutter wegen Mordversuchs an Tochter https://www.spiegel.de/panorama/justiz/hamburg-lange-haft-fuer-mutter-wegen-mordversuchs-an-tochter-mit-medikamenten-a-2293b586-b6d1-4788-89ba-484bc2296d44 Accessed 10 January 2025

[CR105] Spiegel (2021c) Berlinerin muss nach Mordversuch mehr als zehn Jahre ins Gefängnis https://www.spiegel.de/panorama/justiz/berlin-zehneinhalb-jahre-haft-fuer-55-jaehrige-nach-mordversuch-mit-schwermetall-thallium-a-52cbdd09-4cec-4631-953d-edd3774cc53c Accessed 10 January 2025

[CR106] Spiegel (2022a) Lebenslange Freiheitsstrafe für Krankenpfleger wegen versuchter Morde https://www.spiegel.de/panorama/justiz/saarbruecken-krankenpfleger-wegen-versuchter-morde-zu-lebenslanger-haft-verurteilt-a-6bb99917-c2e7-4301-b02e-47f651acb12f Accessed 10 January 2025

[CR107] Spiegel (2022b) Vater vergiftet – Frau zu lebenslanger Haft verurteilt https://www.spiegel.de/panorama/justiz/traunstein-frau-nach-vergiftung-ihres-vaters-zu-lebenslanger-haft-verurteilt-a-cf8fa6f2-1ce9-4769-a1d8-7fab9dd28ed3 Accessed 10 January 2025

[CR108] Spiegel (2022c) Baby wochenlang von Mutter vergiftet https://www.spiegel.de/panorama/familiendrama-baby-wochenlang-von-mutter-vergiftet-a-199093.html Accessed 10 January 2025

[CR109] Spiegel (2023a) Charité-Kardiologe wegen zweifachen Mordes angeklagt https://www.spiegel.de/panorama/justiz/berlin-charite-kardiologe-wegen-zweifachen-mordes-angeklagt-a-64c6629e-3272-4376-96cd-a613c7c4e812 Accessed 10 January 2025

[CR110] Spiegel (2023b) Versuchter Mord mit Rattengift – sechseinhalb Jahre Haft für Musiker https://www.spiegel.de/panorama/justiz/hannover-versuchter-mord-mit-rattengift-sechseinhalb-jahre-haft-fuer-musiker-a-3ba77067-d451-45ff-b103-a97479eb22e4 Accessed 10 January 2025

[CR111] Spiegel (2023c) „Ich habe sie ruhigstellen wollen“ https://www.spiegel.de/panorama/justiz/muenchen-krankenpfleger-gesteht-vor-gericht-nicht-indizierte-medikamentengabe-a-3bc82b20-75bd-4ec1-b994-b0f23b7642df Accessed 10 January 2025

[CR112] Spiegel (2024a) Vater gesteht, Tochter Quecksilber gespritzt zu haben https://www.spiegel.de/panorama/justiz/hannover-mann-gesteht-tochter-quecksilber-gespritzt-zu-haben-a-4953516e-62bc-4b8b-a866-d47a04433d86 Accessed 10 January 2025

[CR113] Spiegel (2024b) Mord mit hochgiftigem Blauen Eisenhut – lebenslange Freiheitsstrafe für Ehemann https://www.spiegel.de/panorama/justiz/dessau-rosslau-mord-mit-hochgiftigem-blauen-eisenhut-lebenslange-freiheitsstrafe-fuer-ehemann-a-950f7886-0590-488d-ac89-888defde3fa9 Accessed 10 January 2025

[CR114] Statista (2025a) Einwohnerzahl in Bayern von 1960 bis 2023. https://de.statista.com/statistik/daten/studie/154879/umfrage/entwicklung-der-bevoelkerung-von-bayern-seit-1961/ Accessed 14 January 2025

[CR115] Statista (2025b) Einwohnerzahl in Brandenburg von 1961 bis 2023. https://de.statista.com/statistik/daten/studie/155142/umfrage/entwicklung-der-bevoelkerung-von-brandenburg-seit-1961/ Accessed 14 January 2025

[CR116] Statista (2025c) Einwohnerzahl in Nordrhein-Westfalen von 1960 bis 2023 https://de.statista.com/statistik/daten/studie/155156/umfrage/entwicklung-der-bevoelkerung-von-nordrhein-westfalen-seit-1961/ Accessed 14 January 2025

[CR117] Statista (2025d) Einwohnerzahl in Rheinland-Pfalz von 1960 bis 2023 https://de.statista.com/statistik/daten/studie/155158/umfrage/entwicklung-der-bevoelkerung-von-rheinland-pfalz-seit-1961/ Accessed 14 January 2025

[CR118] Statista (2025e) Durchschnittsalter der Bevölkerung in Deutschland nach Bundesländern im Jahr 2023 https://de.statista.com/statistik/daten/studie/1093993/umfrage/durchschnittsalter-der-bevoelkerung-in-deutschland-nach-bundeslaendern/ Accessed 14 January 2025

[CR119] Statistisches Bundesamt (Destatis) (2025a) Bevölkerung nach Nationalität und Bundesamt, https://www.destatis.de/DE/Themen/Gesellschaft-Umwelt/Bevoelkerung/Bevoelkerungsstand/Tabellen/bevoelkerung-nichtdeutsch-laender.html Accessed 14 January 2025

[CR120] Statistisches Bundesamt (Destatis) (2025b) Demografischer Wandel https://www.destatis.de/DE/Themen/Querschnitt/Demografischer-Wandel/_inhalt.html#408308 Accessed 15 January 2025

[CR121] Statistisches Landesamt Baden-Württemberg (2024) Bevölkerung nach Altersgruppen und Geschlecht. https://www.statistik-bw.de/BevoelkGebiet/Alter/LRt0104.jsp Accessed 14 January 2025

[CR122] Süddeutsche Zeitung (2014) Versuchter Mord bei der Geburt, https://www.sueddeutsche.de/muenchen/versuchter-mord-in-muenchner-klinik-polizei-nimmt-hebamme-fest-1.2061176 Accessed 08 January 2025

[CR123] Süddeutsche Zeitung (2018) Zehn Jahre Haft für Mordversuch mit Blutverdünner, https://www.sueddeutsche.de/bayern/prozess-in-passau-zehn-jahre-haft-fuer-mordversuch-mit-blutverduenner-1.4063535 Accessed 08 January 2025

[CR124] Süddeutsche Zeitung (2020) Lebenslange Haft wegen Giftmord und Urnenklau. https://www.sueddeutsche.de/muenchen/muenchen-gericht-lebenslange-haft-giftmord-urnenklau-1.5152393 Accessed 08 January 2025

[CR125] Süddeutsche Zeitung (2023) Tötete Altenpfleger aus Geltungsdrang? Angeklagter schweigt. https://www.sueddeutsche.de/panorama/landgericht-bremen-toetete-altenpfleger-aus-geltungsdrang-angeklagter-schweigt-dpa.urn-newsml-dpa-com-20090101-231031-99-774878 Accessed 08 January 2025

[CR126] Universität Bielefeld (2025) Geschichte der Gleichstellung – Chronik https://www.uni-bielefeld.de/uni/profil/gleichstellung/nachgelesen/geschichte-gleichstellung/zeittafel/ Accessed 15 January 2025

[CR127] Várady J (1943) Fall einer akuten Zinkchlorid-Vergiftung mit tödlichem Ausgang. In: Behrens, B. (eds) Sammlung von Vergiftungsfällen. Springer, Berlin, Heidelberg. 10.1007/978-3-662-32714-2_1

[CR128] Verbraucherzentrale (2024) Schwer gefährlich: Giftige Schwermetalle https://www.verbraucherzentrale.de/wissen/lebensmittel/nahrungsergaenzungsmittel/schwer-gefaehrlich-giftige-schwermetalle-13363 Accessed 15 January 2025

[CR129] Vogler U (2018) "Alles hanebüchen" – Bauer will Eltern mit Rattengift umbringen und tischt absurde Erklärungen auf, STERN, https://www.stern.de/panorama/verbrechen/rattengift-anschlag--landwirt-tischt-richter--hanebuechene--story-auf-7884690.html Accessed 04 January 2025

[CR130] Wahrig B (2025) Christa Lehmann trifft Ernst Klee. Technische Universität Braunschweig, https://www.tu-braunschweig.de/pharmaziegeschichte/bibliothek/geschichten-aus-der-bibliothek/christa-lehmann-trifft-ernst-klee Accessed 14 January 2025

[CR131] Wikipedia authors (2025) Der Spiegel. Wikipedia https://de.wikipedia.org/wiki/Der_Spiegel Accessed 14 January 2025

[CR132] ZEIT (1967) Sex, Gift und Geld https://www.zeit.de/1967/47/sex-gift-und-geld Accessed 10 January 2025

[CR133] ZEIT (2021a) Mordversuche mit Medikamenten? – Zwei Prozesse in Bayern https://www.zeit.de/news/2021-09/29/mordversuch-an-frau-mit-medikamenten-angeklagter-schweigt Accessed 10 January 2025

[CR134] ZEIT (2021b) Angeklagte bestreiten Mordversuch mit Gift im Gefängnis https://www.zeit.de/news/2021-04/13/prozess-um-mordversuch-mit-rattengift-im-gefaengnis Accessed 10 January 2025

[CR135] ZEIT (2021c) Versuchter Giftmord: Mehr als sechs Jahre Haft https://www.zeit.de/news/2021-07/08/versuchter-giftmord-mehr-als-sechs-jahre-haft Accessed 10 January 2025

[CR136] ZEIT (2022) Frau soll Lebensgefährten über längere Zeit vergiftet haben https://www.zeit.de/news/2022-10/06/frau-soll-lebensgefaehrten-ueber-laengere-zeit-vergiftet-haben Accessed 10 January 2025

[CR137] ZEIT (2023) Prozess um mutmaßlichem Giftmord startet im Juni https://www.zeit.de/news/2023-05/25/prozess-um-mutmasslichem-giftmord-startet-im-juni Accessed 10 January 2025

