# Supplementary material for: Systematic analysis of homicidal poisonings in Germany
Source: Naunyn Schmiedebergs Arch Pharmacol. 2025 Dec 13;399(5):7547–80. doi: 10.1007/s00210-025-04823-3 (PMC13053436; doi:10.1007/s00210-025-04823-3)

**Systematic analysis of homicidal poisonings in Germany**

**Madeleine Ohm and Roland Seifert**

Supplemental Figures

***Figure S1:*** *The distribution of the selected articles across the four newspapers, shown in a pie chart.*

***
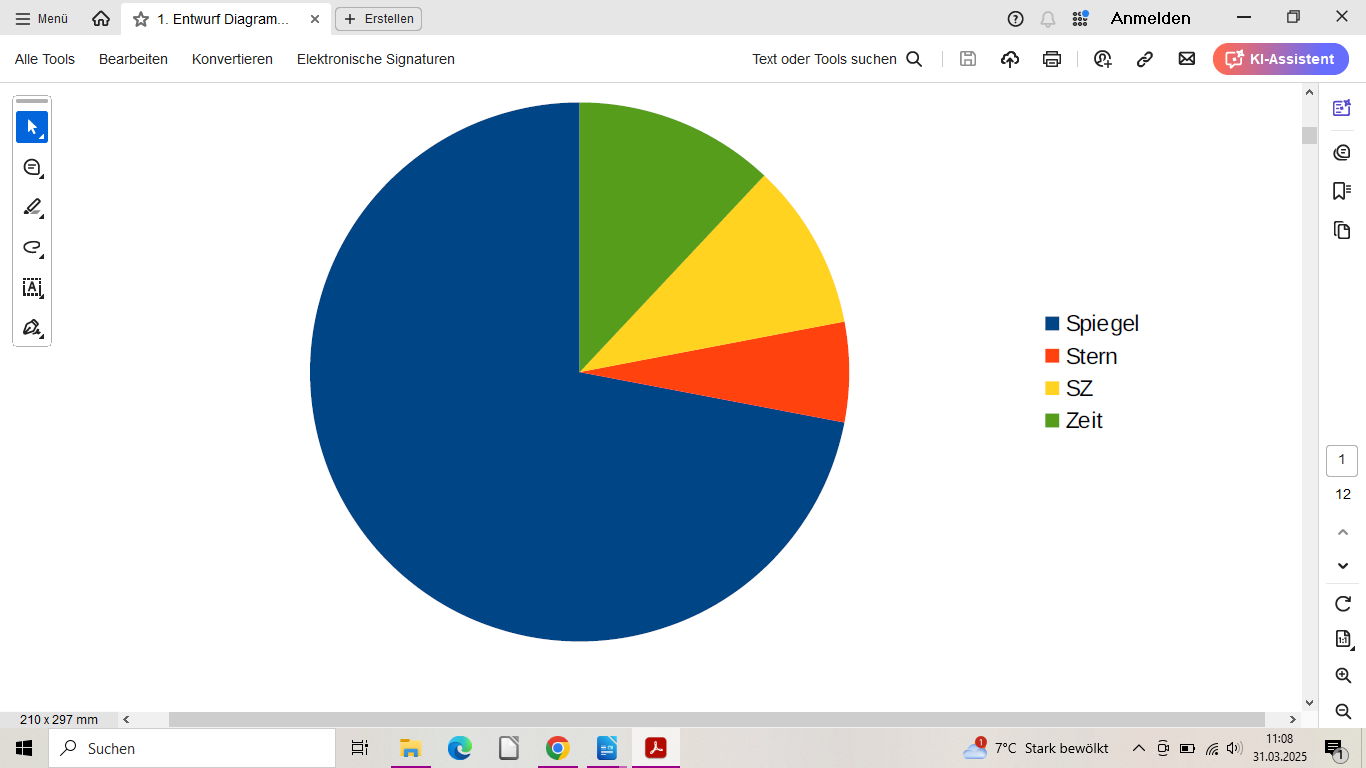
***

***Figure S2:*** *The genders of the perpetrators, shown in a bar chart in which the total value (*percentage value of all perpetrators) is blue, the value before the reunion is red and the value after the reunion is yellow.*


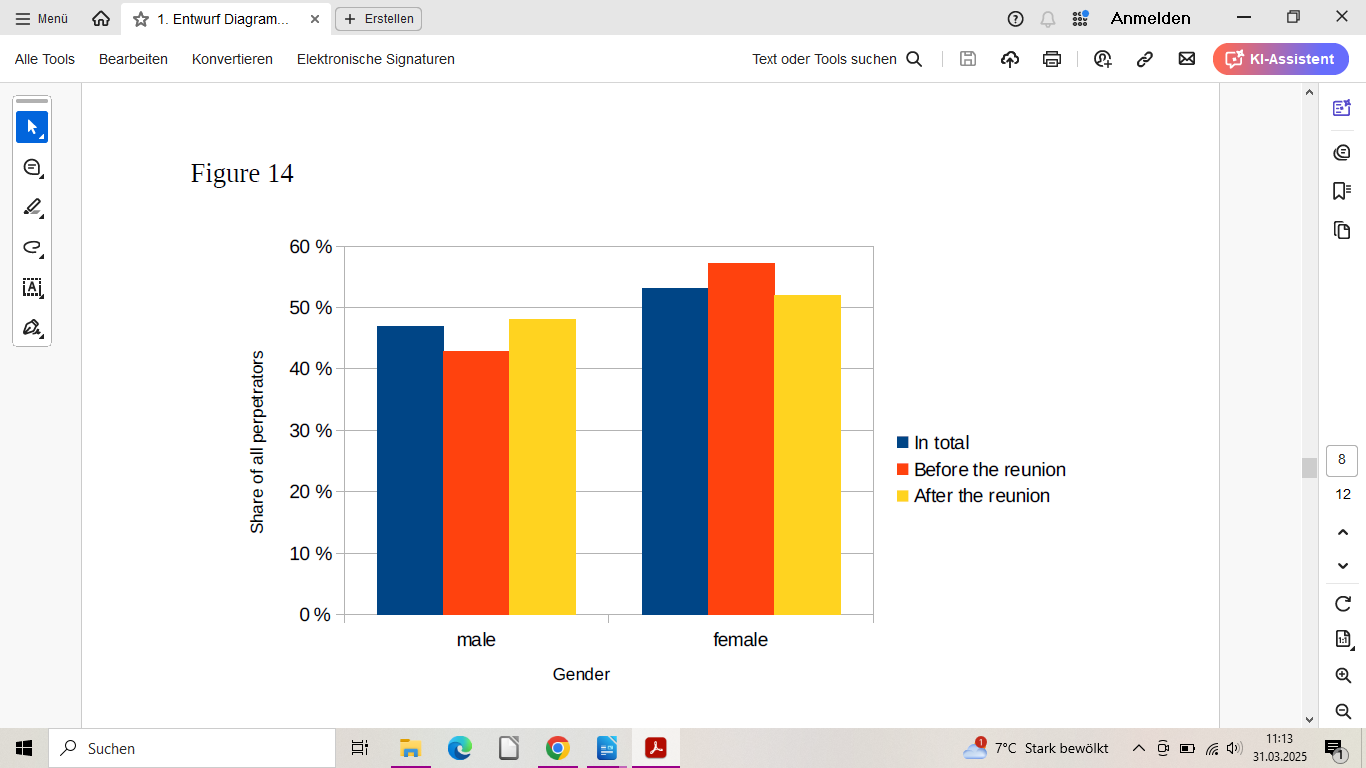


***Figure S3:*** *The genders of the victims, shown in a bar chart in which the total value (*percentage value of all victims) is blue, the value before the reunion is red and the value after the reunion is yellow.*


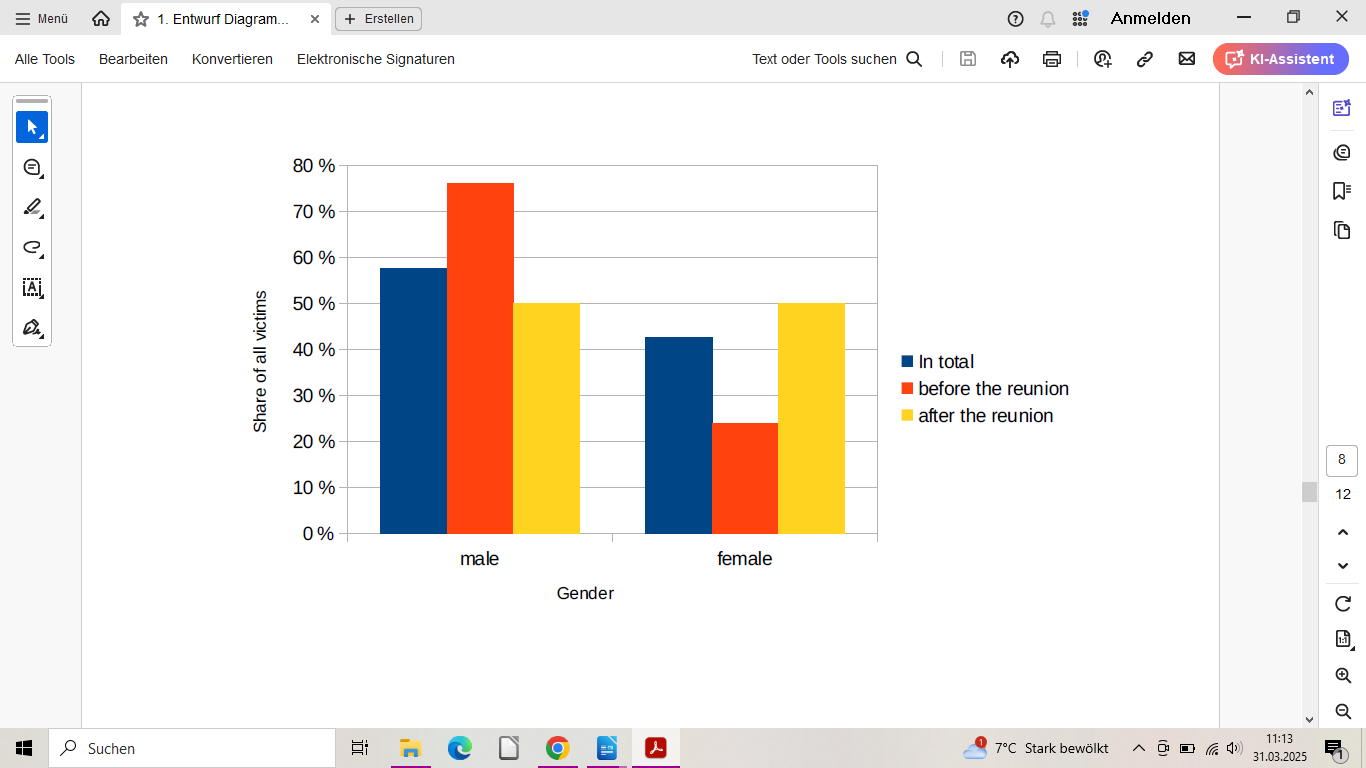


***Figure S4:*** *How many victims the perpetrators poisoned or tried to poison, shown in a bar chart in which the total value (*percentage value of all perpetrators) is blue, the value before the reunion is red and the value after the reunion is yellow.*


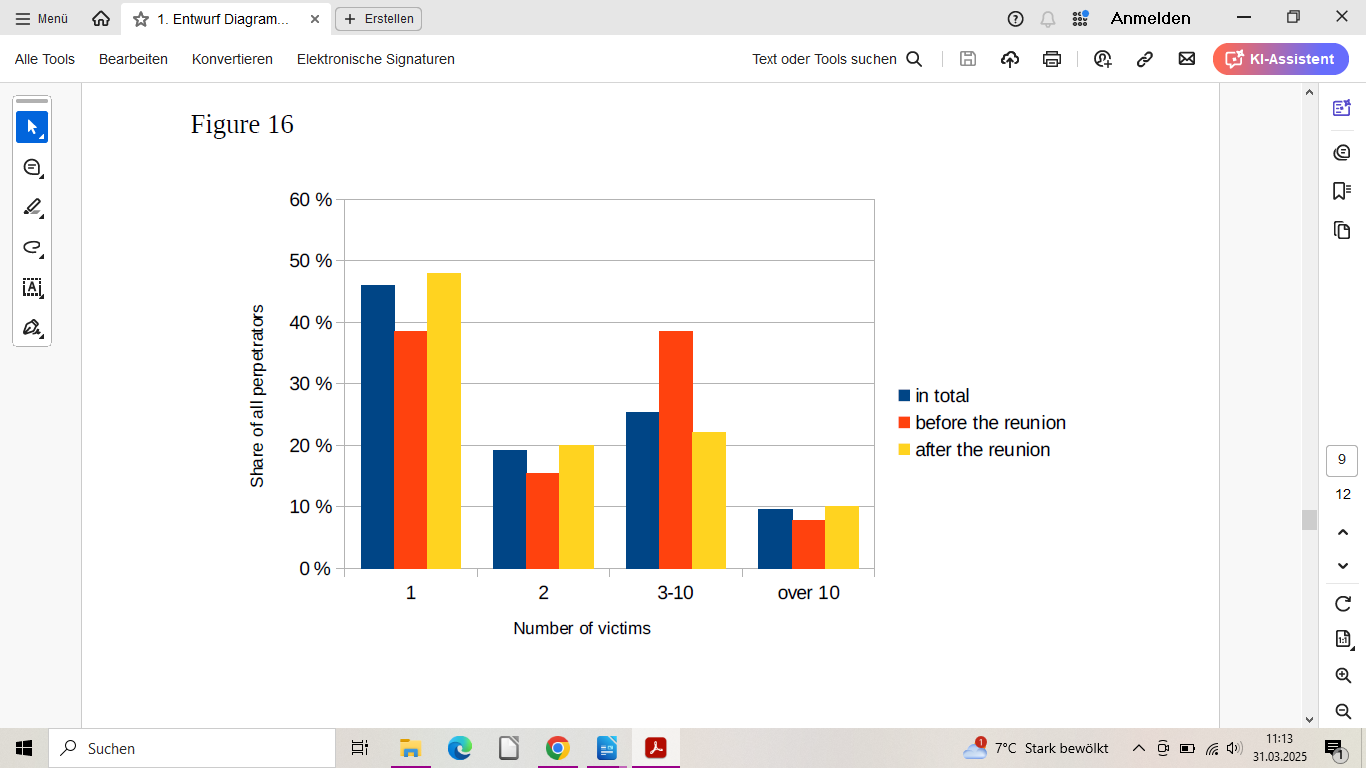


***Figure S5:*** *The relationship of victim and perpetrator presented in the role the perpetrator had, shown in a bar chart in which the total value (*percentage value of all perpetrators) is blue, the value before the reunion is red and the value after the reunion is yellow.*

*
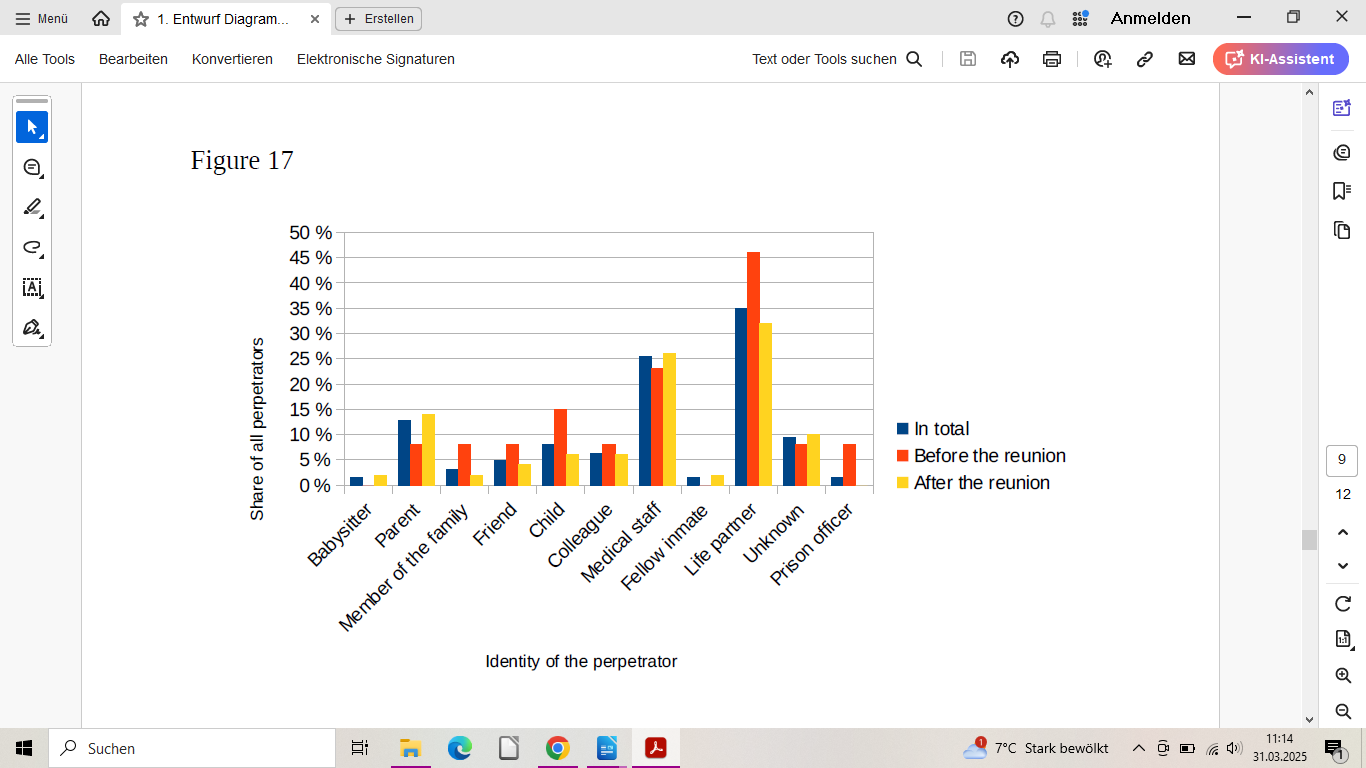
*

***Figure S6:*** *Where or how the perpetrators got the poison they used, shown in a bar chart in which the total value (*percentage value of all perpetrators) is blue, the value before the reunion is red and the value after the reunion is yellow.*


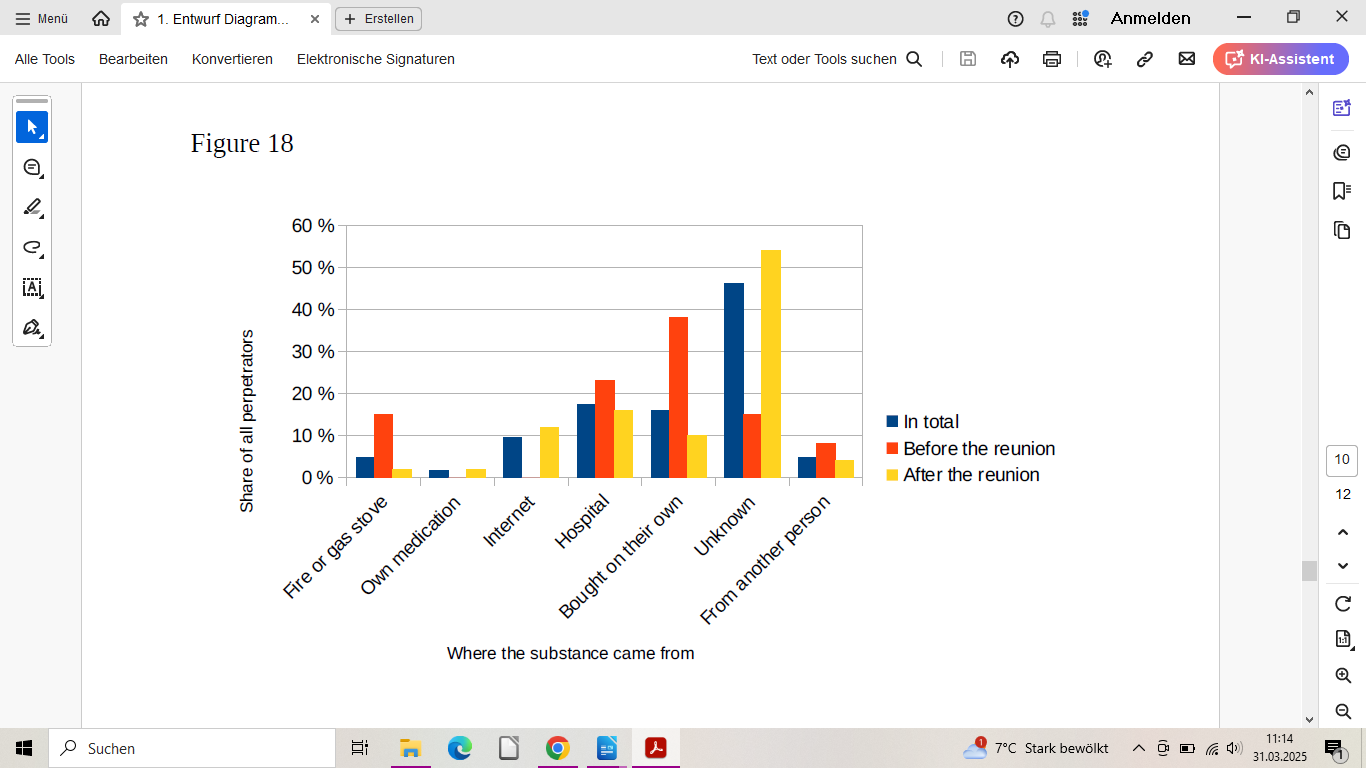


***Figure S7:*** *The motives that lead to the perpetrators poisoning their victims, shown in a bar chart in which the total value (*percentage value of all perpetrators) is blue, the value before the reunion is red and the value after the reunion is yellow.*


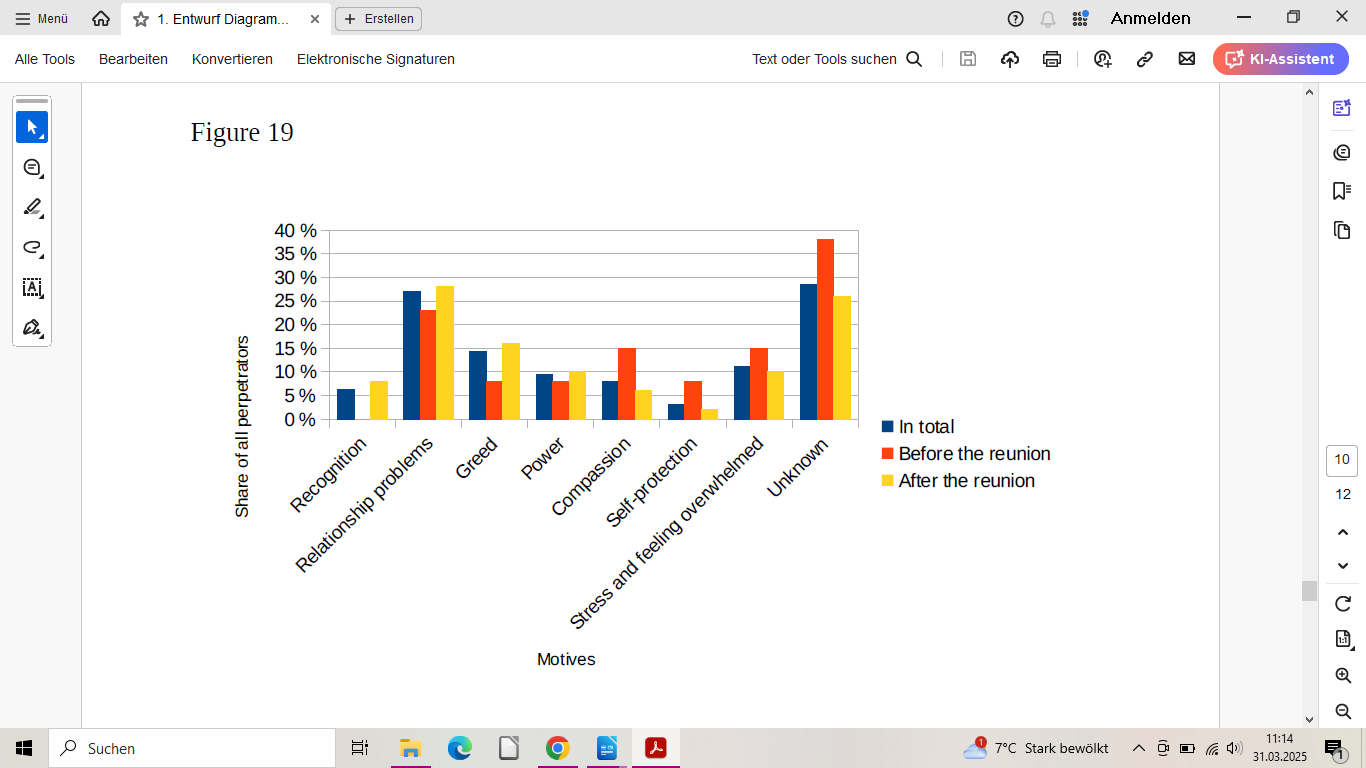


***Figure S8:*** *By whom or how it was noticed that the victim got poisoned, shown in a bar chart in which the total value (*percentage value of all cases) is blue, the value before the reunion is red and the value after the reunion is yellow.*


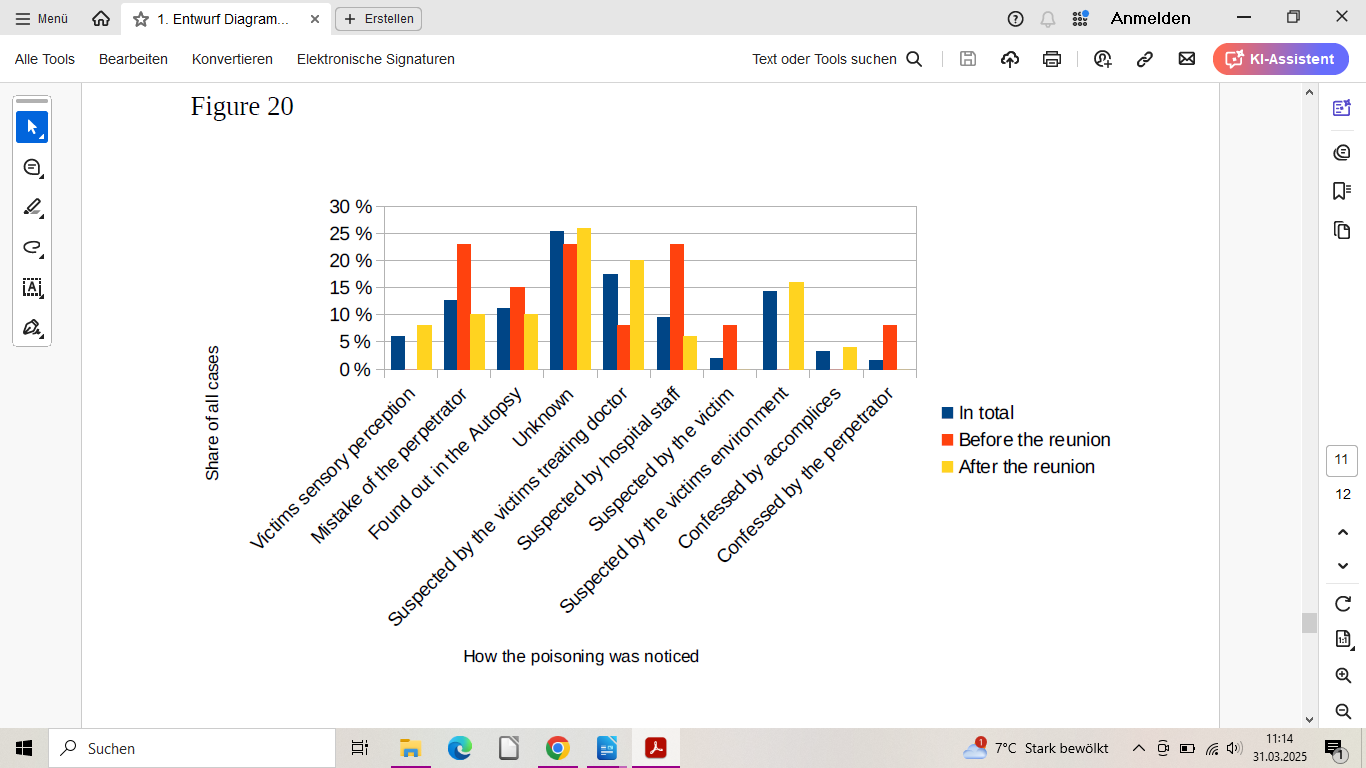


***Figure S9:*** *The court rulings the perpetrators got, shown in a bar chart in which the total value (*percentage value of all perpetrators) is blue, the value before the reunion is red and the value after the reunion is yellow.*

*
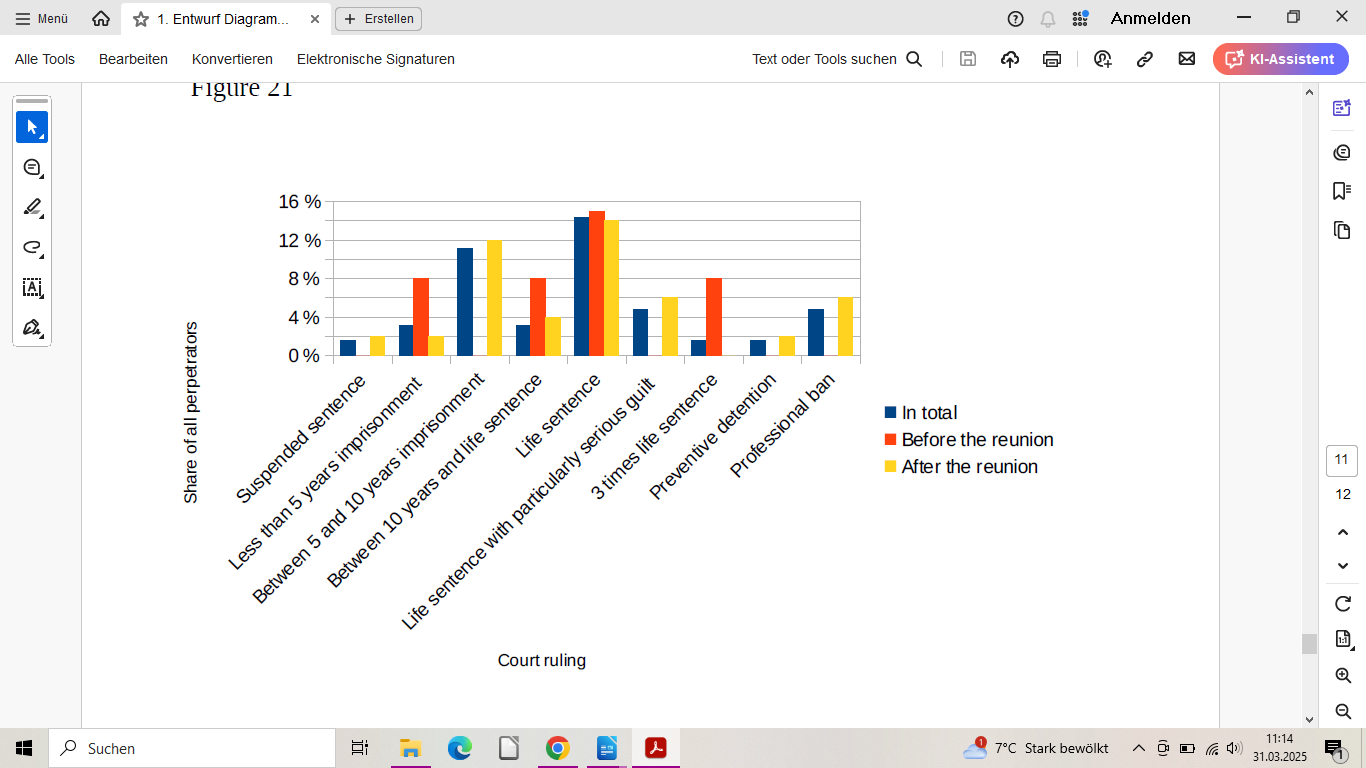
*

***Figure S10:*** *Crimes against life per 1,000,000 inhabitants in each federal state in 2023, shown in a bar chart.*


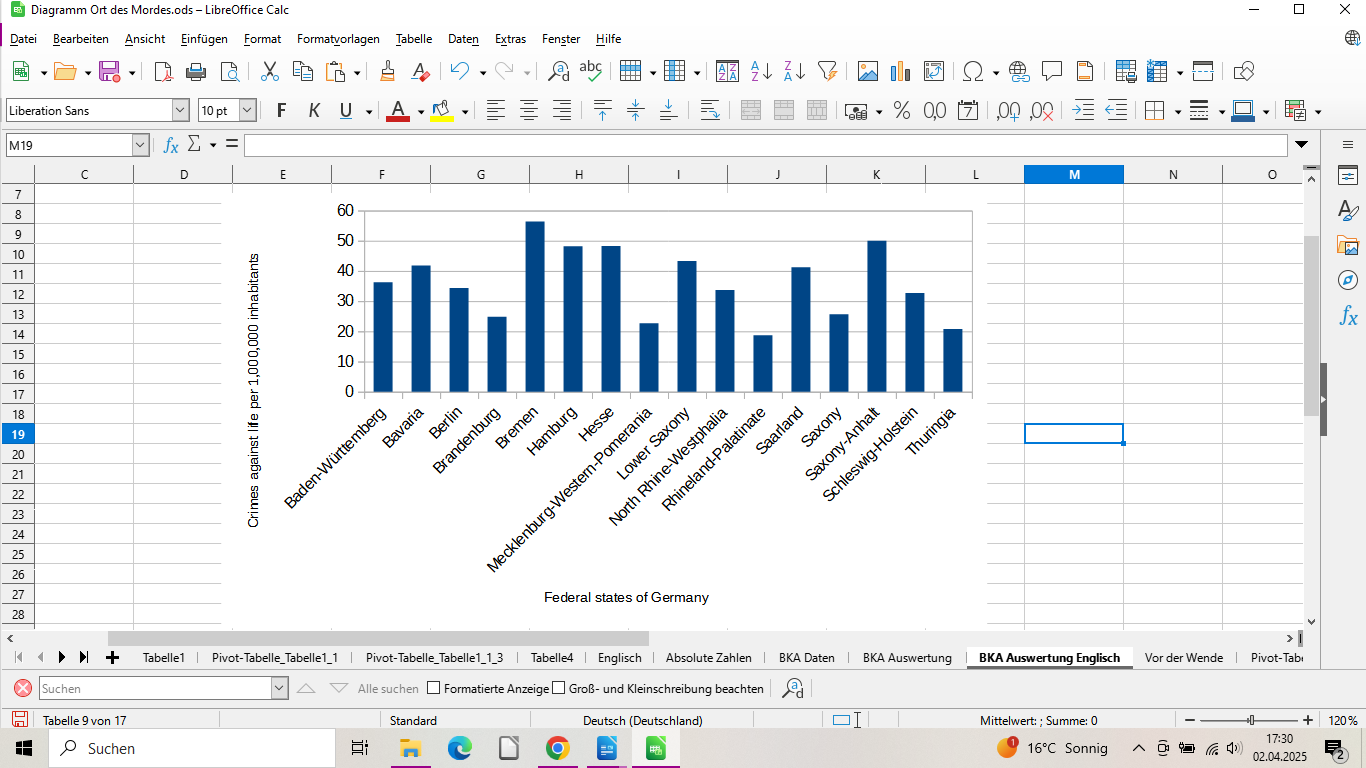

Supplement: Supplementary file 1 — (DOCX 1.29 MB) [file 210_2025_4823_MOESM1_ESM.docx]
